# Chronic Cerebral Hypoxia and Cognitive Impairment: A Systematic Review and Meta‐Analysis Based on Chronic Mountain Sickness, Anemia, Chronic Obstructive Pulmonary Disease, and Obstructive Sleep Apnea

**DOI:** 10.1002/cns.70875

**Published:** 2026-04-16

**Authors:** Haishi Fei, Guirong Cheng, Yan Zeng, Feibo Zhao, Zhichao He, Shengzhong Yi

**Affiliations:** ^1^ Brain Science and Advanced Technology Institute, School of Medicine Wuhan University of Science and Technology Wuhan China; ^2^ Hubei Provincial Clinical Research Center for Alzheimer's Disease, Tian You Hospital Affiliated to Wuhan University of Science and Technology Wuhan University of Science and Technology Wuhan China; ^3^ Hubei, Cancer Hospital, Tongji Medical College Huazhong University of Science and Technology Wuhan China

**Keywords:** anemia, chronic hypoxia, chronic mountain sickness (CMS), chronic obstructive pulmonary disease (COPD), cognitive function, obstructive sleep apnea (OSA)

## Abstract

**Background:**

Chronic hypoxia, a key pathological feature of chronic mountain sickness (CMS), anemia, Chronic Obstructive Pulmonary Disease (COPD), and Obstructive Sleep Apnoea (OSA), impairs cognitive function; however, their association strength, shared mechanisms, and disease‐specific differences remain unsystematized, hindering early interventions.

**Objective:**

This study aimed to quantify these via a systematic review and meta‐analysis to clarify the deficits and mechanisms for clinical guidance.

**Methods:**

We searched PubMed, Web of Science, Embase, and Cochrane Library for relevant studies, assessed the quality using the Newcastle‐Ottawa Scale, and analyzed the data using Stata 18.0.

**Results:**

Forty‐one studies involving 18 countries, 369, 619 participants (5 on CMS, 8 on anemia, 11 on OSA, and 17 on COPD) demonstrated that all four diseases were associated with an increased risk of cognitive impairment, with OR ranging from 1.370 to 6.892. In dichotomous analyses, anemia was epidemiologically linked to elevated cognitive impairment risk but showed nonsignificant, heterogeneous effects on continuous cognitive scores and no associations with specific cognitive domains, indicating its impact is moderated by population traits and measurement approaches. The other three diseases impaired global and domain‐specific cognition with SMD ranging from −0.6352 to −0.2000, each with unique deficit profiles. Notably, correction for publication bias eliminated the statistical significance of the overall pooled OR for cognitive impairment risk.

**Conclusion:**

Hypoxia is the core shared mechanism linking these four diseases to cognitive impairment, involving mitochondrial dysfunction and neuroinflammation, with additional modulation by genetic and adaptive factors. However, current evidence is limited by publication bias and inconsistent findings (e.g., for anemia). These conclusions must be interpreted with extreme caution, and high‐quality longitudinal studies are needed to confirm causality.

## Graphical abstract

Meta‐analysis of 41 studies revealed chronic hypoxia in conditions such as chronic mountain sickness, anemia, COPD, and OSA significantly increases the risk of cognitive impairment. Shared mechanisms include mitochondrial dysfunction and neuroinflammation, while unique pathways such as erythrocytosis‐induced hyperviscosity and gut–lung–brain axis dysregulation contribute to disease heterogeneity.

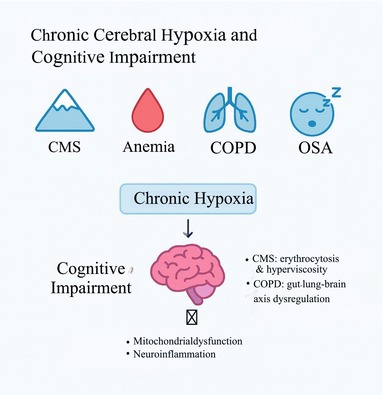

## 1 Introduction

Cognitive function, encompassing memory, attention, and executive function across multiple domains, is integral to quality of life and social functioning [1]. Recent advances in basic and clinical research have highlighted the impact of systemic disease‐induced chronic pathological states on cognition, with chronic hypoxia—a key shared feature of diverse conditions—emerging as a critical risk factor for cognitive impairment. Chronic Mountain Sickness (CMS) [2], anemia [3], Obstructive Sleep Apnea (OSA) [4], and Chronic Obstructive Pulmonary Disease (COPD) [5] are the clinically prevalent chronic hypoxic disorders. Despite distinct pathophysiologies, they may impair cognition through disrupted oxygen transport, gas exchange, or systemic metabolism, leading to cerebral hypoperfusion or neuronal damage.

In CMS, hypoxemia from high‐altitude hypoxia directly impairs cerebral neurons, causing attention deficits and memory decline [6]. Anemia, via reduced hemoglobin oxygen‐carrying capacity, induces cerebral hypoxia that disrupts neurotransmitter synthesis and signaling [7]. OSA‐related recurrent nocturnal apnea and hypoxemia disrupt sleep architecture and damage cognition‐critical regions like the hippocampus [8]. In COPD, chronic hypoxia and carbon dioxide retention from airflow limitation accelerate white matter lesions, elevating cognitive impairment risk [9].

However, current evidence has limitations: most studies focus on single diseases with small samples, lacking cross‐disease comparisons; heterogeneous cognitive assessment tools and hypoxia quantification criteria increase result variability; crucially, shared and disease‐specific mechanisms linking these conditions to cognitive impairment remain poorly integrated, hindering understanding of the “chronic hypoxia‐cognitive impairment” pathway.

This study addresses these gaps via systematic review and meta‐analysis, synthesizing clinical evidence to: (1) quantify overall cognitive impairment risk in CMS, anemia, OSA, and COPD, with cross‐disease comparisons; (2) explore differential effects on global and domain‐specific cognition; and (3) systematically summarize shared and disease‐specific pathophysiological mechanisms. Findings aim to establish evidence‐based standards for the early clinical identification of cognitive impairment in chronic hypoxic diseases and inform cross‐disease cognitive protection strategies targeting cerebral hypoxia.

## 2 Methods

### 2.1 Protocol and Registration

This review strictly adhered to the Preferred Reporting Items for Systematic Reviews and Meta‐Analyses (PRISMA) [10] and Meta‐analysis of Observational Studies in Epidemiology (MOOSE) guidelines [11] to ensure consistency and transparency. It was registered in the International Prospective Register of Systematic Reviews (CRD420251106030).

### 2.2 Literature Search

A comprehensive search was conducted in PubMed, Web of Science, Embase, and Cochrane Library, covering publications from database inception to June 30, 2025. Reference lists of relevant reviews were also hand‐searched. Search terms included exposure factors (“High Altitude,” “Anemia,” “OSA,” “COPD”) and outcomes (“cognitive impairment,” “cognitive decline,” “mild cognitive impairment,” “cognitive disorder,” “cognitive function”), with synonyms combined using “OR” and variables linked by “AND.” The detailed strategy is provided in (Table S1).

### 2.3 Study Selection

Inclusion criteria: (1) Observational studies (including cohort, cross‐sectional, and case–control studies). (2) Participants were aged ≥ 18 years. (3) Exposure variables related to the four conditions: High Altitude, Anemia, OSA, or COPD. (4) Outcome variables assessing global or domain‐specific cognitive functions or identifying cognitive impairment. (5) Studies providing clear disease diagnostic criteria and sufficient data for association analyses (e.g., raw data, Pearson correlations, standardized means, β coefficients, ORs with 95% CIs). (6) Published in English.

Exclusion criteria: (1) Inclusion of minors; (2) Participants with uncontrolled severe cardio‐hepato‐renal comorbidities or neuropsychiatric disorders (e.g., multiple sclerosis, Parkinson's disease, schizophrenia, and traumatic brain injury); (3) Outcomes unrelated to cognitive function/impairment; (4) Overt acute hypoxia (e.g., acute mountain sickness); and (5) Study type (reviews, randomized controlled trials, case reports, animal studies, letters, editorials, conference abstracts, or duplicates).

### 2.4 Operational Definition

To enhance comparability, the core variables were defined as follows:

Exposure variables: CMS: Residence at ≥ 2, 500 m for ≥ 6 months [12]; Anemia: WHO criteria (hemoglobin < 13 g/dL in males, < 12 g/dL in females) [13]; OSA: Apnea‐hypopnea index (AHI) ≥ 5 events/h (vs. AHI < 5 for non‐OSA) [14]; COPD: Post‐bronchodilator FEV_1_/FVC < 0.7 with chronic respiratory symptoms (per GOLD/ATS/ERS guidelines) [15, 16] and auxiliary criteria (e.g., medical record codes, ≥ 1‐year duration) for specific populations [17]. The “positive hypoxia indicator” subgroup was defined based on objective oxygenation parameters: arterial partial pressure of oxygen (PaO_2_) < 80 mmHg or oxygen saturation (SpO_2_) < 92% at rest, combined with disease severity classification (moderate‐to‐severe OSA/COPD, severe anemia, or long‐term high‐altitude residence ≥ 3000 m).

Outcome variables: The primary outcomes are cognitive impairment and cognitive function, as defined below.

Cognitive impairment: Encompassing dementia, mild cognitive impairment (MCI), and other deficits defined via standardized scale cutoffs (e.g., MMSE < 24, MoCA < 26) [15, 18], subjective complaints, objective neuropsychological tests, functional assessments, and exclusion of reversible causes. “Other deficits” included scores < 15th percentile (or 1 SD below mean) in neuropsychological tests without explicit dementia/MCI labeling.

Cognitive domains: Seven core domains [19] (executive function, memory, processing speed, language, attention‐working memory, visuospatial ability, and global cognition) derived from 85 tasks (Table S2). Two reviewers (H. Fei, Z. He) independently coded domains; inter‐coder consistency (Kappa ≥ 0.8) was verified, with discrepancies resolved by a third reviewer (G. Cheng).

### 2.5 Data Extraction and Quality Assessment

Three reviewers (H. Fei, F. Zhao, Z. He) independently extracted data from eligible studies, including: study origin, location, publication year; participant characteristics (age, sample size, sex distribution); diagnostic criteria, outcome assessment methods, adjusted covariates; and quantitative data (e.g., mean ± SD of cognitive scores, impairment prevalence) for pooled effect size calculations. Data were cross‐checked, with discrepancies resolved via team discussion or arbitration by a fourth reviewer.

Study quality was assessed using the 9‐item Newcastle‐Ottawa Scale (NOS) adapted for the study type, covering three key domains: selection of study population, comparability of groups, and assessment of outcomes. Two reviewers independently rated quality, with discrepancies resolved by a third. Quality categories were defined by cutoffs: high quality (≥ 7 points), moderate quality (5–6 points), and low quality (≤ 4 points) [20].

### 2.6 Statistical Analyses

Pooled results were presented as odds ratios (ORs) and standardized mean differences (SMDs) with 95% CIs. Model selection depended on heterogeneity: fixed‐effects model for *I* ^2^ ≤ 50% and *p* ≥ 0.05; otherwise, random‐effects model. The most fully adjusted was selected for studies with multiple adjusted values.

Traditional meta‐analytic models assume independent effect sizes [21], but multiple independent effect sizes were allowed here because of (1) multi‐tool assessments of the same cognitive construct; (2) multiple outcomes; and (3) time‐point variations [22]. Independence within studies was not assumed to avoid overestimating correlations [23].

For cognitive impairment data, a two‐level meta‐analytic model recalculated effect sizes for multiple outcomes/exposures. Heterogeneity was assessed using *Q* and *I* ^2^ statistics, and subgroup analyses were used to identify the sources. Publication bias was evaluated using Egger's test with trim‐and‐fill adjustment if needed. Leave‐one‐out sensitivity analyses were used to assess individual study impacts. Additionally, to verify the robustness of the pooled effect size, an additional sensitivity analysis was performed by excluding potentially small‐sample studies (*n* < 100) and studies with moderate‐to‐low methodological quality (Newcastle‐Ottawa Scale score ≤ 6). All analyses used Stata 18.0 (Stata Corp).

For cognitive function scores, a three‐level model (“study → cognitive domain → tool”) controlled for multi‐level variability (e.g., between‐study heterogeneity, within‐study domain differences) to avoid bias. Compared with the traditional two‐level model, it is more appropriate for this nested data structure (tools within cognitive domains within studies), as it captures nested variability overlooked by the two‐level model. Model convergence and estimation robustness were ensured via restricted maximum likelihood estimation and log‐likelihood ratio tests (comparing the three‐level model with variance‐excluded models [24]). Parameters were estimated via multilevel random‐effects models with restricted maximum likelihood using the R software (R Foundation for Statistical Computing, Vienna, Austria).

## 3 Results

### 3.1 Study Selection and Characteristics

**FIGURE 1:**
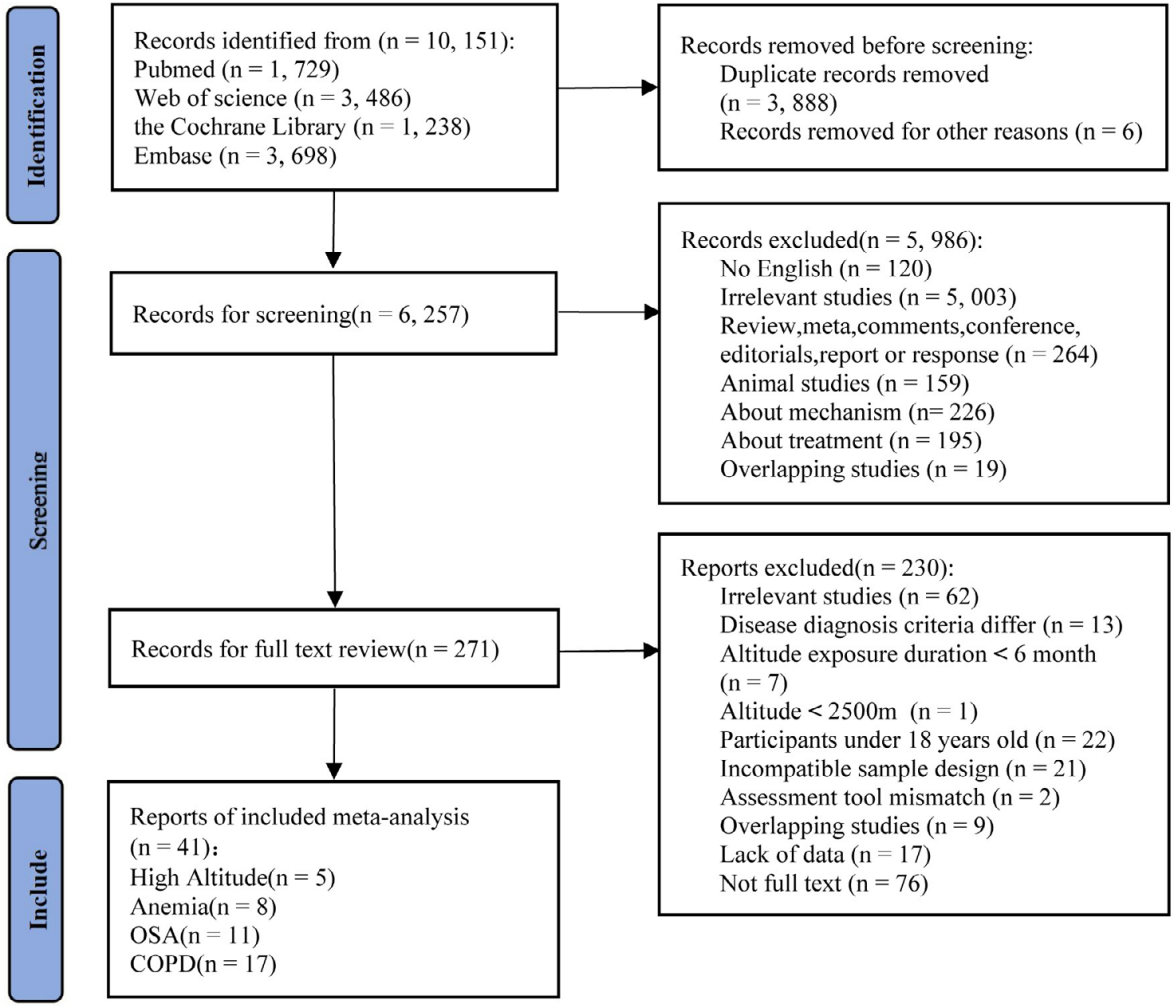
Flow chart of manuscript selection process.

A comprehensive search identified 10, 151 records. After duplicate removal, 271 potentially relevant studies underwent full‐text review, with 41 ultimately meeting inclusion criteria (Figure 1) [13, 14, 15, 16, 17, 25, 26, 27, 28, 29, 30, 31, 32, 33, 34, 35, 36, 37, 38, 39, 40, 41, 42, 43, 44, 45, 46, 47, 48, 49, 50, 51, 52, 53, 54, 55, 56, 57, 58, 59, 60]. These studies spanned 18 countries, comprising 41 cohort studies with 369, 619 participants. Sample sizes varied markedly 42 [39] to 307, 817 [17], and median ages ranged widely 19.16 years [48] to 77.42 years [59] (Table 1).

**TABLE 1:** Study characteristics.

Disease	Study id	Authors; year	Country	Cases (*n*)	Controls (*n*)	Age (mean)	Males (%)	Disease diagnostic criteria	Cognitive impairment tool	Cognitive impairment criteria	Adjusted confounding factors^a^	Hypoxia indicators	NOS
CMS	1	Chen; 2019	China	49	49	19.16	65.30	Average altitude 3658 m for 280.8 ± 7.3 days	ACRT; DVBM; IVBM; ASRT	NA	NA	NA	8
	2	Das; 2018	India	153	205	30.00	100.00	Altitude of 4500–4800 m for 12 months	MMSE; MDCST; MDCST	NA	NA	SpO_2_	6
	3	Gao; 2015	China	217	141	21.68	100.00	Altitude of 4500 m for 1–5 years	RSPM; Digital span test; Visual reaction time; Audible reaction time; Digital symbol test; Benton visual retention test; Pursuit aiming test	NA	NA	NA	7
	4	Hota; 2012	India	843	862	36.30	100.00	Altitude above 4300 m for more than 12 months	MMSE; MDCST	The MMSE or MDCST total score < 25	NA	NA	7
	5	Zhang; 2022	India	49	43	31.81	42.40	Altitude above 4300 m	MoCA	The MoCA total score	NA	NA	6
Anemia	6	Beydoun; 2020	United States	262	1371	46.90	45.00	Anemia as Hb < 13 g/dL for men and < 12 g/dL for women	MMSE	NA	NA	NA	8
	7	Deal; 2009	United States	33	341	74.00	0.00	Anemia as < 12 g/dL for women	HVLT‐immediate recall; HVLT‐delayed recall; TMT‐A; TMT (B‐A)	NA	Sociodemographic; Health‐related behavioral; Clinical conditions	NA	8
	8	Dlugaj; 2016	Germany	163	3870	64.40	50.00	Anemia as Hb < 13 g/dL for men and < 12 g/dL for women	8—Word List from The Nuremberg Geriatric Inventory; Animal semantic fluency test	The current International Working Group on MCI criteria	Sociodemographic; Health‐related behavioral; Clinical conditions; Genetic factors	NA	8
	9	Karismaz; 2024	Turkey	190	350	77.42	0.00	Anemia as < 12 g/dL for women	MMSE	NA	Clinical conditions	NA	5
	10	Marzban; 2021	Iran	223	2777	70.93	51.73	Anemia as Hb < 13 g/dL for men and < 12 g/dL for women	Mini‐Cog or the Category Fluency Test	An abnormal score on either the Mini‐Cog or the Category Fluency Test	Sociodemographic; Health‐related behavioral; Clinical conditions	NA	7
	11	Qin; 2019	China	1404	9513	59.13	58.81	Anemia as Hb < 13 g/dL for men and < 12 g/dL for women	Episodic memory and TICS score	NA	Sociodemographic; Health‐related behavioral; Clinical conditions	NA	7
	12	Valladão; 2020	Brazil	713	12,911	51.60	45.20	Anemia as Hb < 13 g/dL for men and < 12 g/dL for women	Consortium to Establish a Registry for Alzheimer's Disease word memory test; Animal semantic fluency test; TMT‐B	NA	Sociodemographic; Health‐related behavioral; Clinical conditions	NA	7
Anemia	13	Zamboni; 2006	Italy	5135	8166	72.00	50.00	Anemia as Hb < 13 g/dL for men and < 12 g/dL for women	Abbreviated mental test	Abbreviated mental test score < 7	Sociodemographic; Health‐related behavioral; Clinical conditions	NA	6
OSA	14	Badr; 2023	Egypt	30	20	44.96	56.02	OSA patients who met the diagnostic criteria for AHI according to the International Classification of Sleep Disorders	MMSE; MoCA; BKSCA; D2 Test; Wisconsin Card Sorting Test	NA	NA	SaO_2_ nadir	6
	15	Borges; 2013	Brazil	22	22	51.40	59.10	Moderate to severe OSAS using the American Academy of Sleep Medicine Task Force (1999) guidelines (AHI > 15)	Conners Continuous Performance test; Corsi Blocks Forward; Digit Span Forward; Counting Span; Stroop; Dual task; Trail Making Part B‐A; Plus minus; Zoo Map; Letter memory; Phonemic fluency; Semantic fluency	NA	NA	Percentage total sleep time with SaO_2_ < 90%	7
	16	Buratti; 2017	Italy	34	34	65.57	84.05	Patients with an AHI between 15 and 30 were considered to have moderate OSAS; patients with an AHI greater than 30 had severe OSAS	MMSE; Digit Span; Stroop Test; Rey Figure; Rey Auditory Verbal Learning Test; Verbal Fluency; Phonetic Fluency; Progressive Raven Matrices; Corsi Cubes	NA	NA	ODI; AD SaO_2_	7
	17	Cavuoto; 2023	Australia	35	12	57.76	53.19	The absence of any sleep‐disordered breathing (AHI < 5); OSA (AHI > 10 events/h)	MMSE; Logical Memory Delayed Recall; Rey Osterreith Complex Figure Test Delayed Recall; Autobiographical Memory Test; Digits backward; Trail Making Test (B‐A); Groton Maze Learning; Letter Fluency; Category Fluency; Digit Symbol Coding; Detection Speed; Identification Speed	NA	NA	ODI 3%	7
	18	Chen; 2011	China	348	46	46.10	92.73	Primary snoring group (AHI < 5 events/h). mild OSAHS group (AHI 5–20 events/h); moderate OSAHS group (AHI > 20–40 events/h); severe OSAHS group (AHI > 40 events/h)	MMSE; MoCA	The MoCA score of < 26	NA	Time (SaO_2_ < 90%); Minimum SaO_2_; ODI	6
OSA	19	Kim; 2017	South Korea	781	711	60.39	47.72	No OSA (AHI < 5) and OSA (AHI ≥ 5)	Verbal Fluency‐Phonemical; Verbal Fluency‐Categorical; Digit Symbol‐Coding; TMT‐A; Stroop Test; TMT (B‐A); Visual SaO_2_; Average Reproduction; Logical Memory‐Immediate Recall; Delayed Recall; Recognition	NA	Sociodemographic; Health‐related behavioral; Clinical conditions	SaO_2_, min; SaO_2_, average; ODI	7
	20	Kong; 2021	China	35	16	40.20	86.33	OSAHS was classified as severe (AHI > 30)	MMSE	NA	NA	Mean SaO_2_; Low SaO_2_; ODI	7
	21	Lutsey; 2016	United States	513	442	61.30	45.30	OSA severity groups according to the AHI: < 5.0 events/h (normal); 5.0–14.9 events/h (mild sleep apnea); 15.0–29.9 events/h (moderate sleep apnea); and ≥ 30.0 events/h (severe sleep apnea)	MMSE; TMT‐B; Delayed Word Recall; Logical Memory Test, Part A; Logical Memory Test, Part B; Incidental Learning; Digit‐symbol pairs; Clock Time Perception; TMT‐A; Digit Symbol Substitution; Digit Span Backwards; Word Fluency; Animal Naming; Boston Naming Test	NA	Sociodemographic; Health‐related behavioral; Clinical conditions; Genetic factors	NA	8
	22	Macchitella; 2024	Italy	42	16	51.71	63.79	OSAS was confirmed by recorded an AHI > 5 apnoeas/h of sleep	MoCA	NA	NA	Mean SaO_2_; ODI; Nadir SaO_2_	7
	23	Mekky; 2022	Egypt	764	681	50.71	51.35	According to The AASM Manual for the Scoring of Sleep and Associated Events	MoCA	The MoCA score of < 26	Sociodemographic; Clinical conditions; Premorbid cognition and sleep status	NA	7
	24	Yerlikaya; 2018	Turkey	54	34	37.41	95.45	OSA was classified as severe (AHI > 30)	Oktem verbal memory processes test total learning scores; immediate recall scores	NA	NA	Time with SO_2_ < 90%; Time with SO_2_ < 80%	6
COPD	25	Bratek; 2015	Poland	24	18	58.86	45.24	Standardized interview (exertional dyspnea, progressive, chronic, productive cough); FEV1/FVC < 0.7 after inhalation of bronchodilators	MMSE; TMT‐A; TMT‐B	NA	NA	NA	6
	26	Cleutjens; 2017	Netherlands	90	90	63.00	52.22	Clinically stable COPD, according to the GOLD 2011 strategic document	MMSE; Digit Span Backwards; Behavioral Assessment of the Dysexecutive Syndrome key search; Behavioral Assessment of the Dysexecutive Syndrome zoo map; Stroop Color and Word Test card III; Category Switching Test part C; Visual Verbal Learning Test; Stroop Color and Word Test Card I; Category Switching Test part A; Letter Digit Substitution Test 60 s	Global cognitive impairment was defined as a score of 24 or below on the MMSE	Sociodemographic; Health‐related behavioral; Clinical conditions	PaO_2_; PaCO_2_	8
	27	Crisan; 2014	Romania	39	13	60.71	NA	Patients meet the ATS/ERS criteria for COPD risk class d	MoCA	NA	NA	PaO_2_; PaCO_2_	6
	28	Dodd; 2013	England	80	30	68.18	77.27	Clinically diagnosed COPD patients	WAIS‐III UK Letter‐Number Sequencing; Wechsler Memory Scale‐III UK Spatial Span; Delis‐Kaplan Trail Making Test; Rey Complex Figure Test Recall; Wechsler Memory Scale ‐III UK Word Lists; WAIS‐III Digit Symbol; WAIS‐III Symbol Search; Delis‐Kaplan Verbal Fluency; Rey Complex Figure Test Copy	NA	Sociodemographic; Health‐related behavioral; Clinical conditions	PaO_2_; PaCO_2_	7
	29	Isoaho; 1996	Finland	81	245	71.52	74.23	COPD was diagnosed if the spirogram showed the forced expiratory volume in 1 s as a percentage of the forced vital capacity (FEV1/FVC%) to be equal to or < 65; irrespective of possible reversibility of obstruction	MMSE	Cognitive impairment was defined as present if the total score on the MMSE was 0–23	NA	NA	6
COPD	30	Klein; 2010	Austria	60	60	63.35	60.00	Diagnosis of COPD based on the criteria of the American Thoracic Society	Attention Network Test; Verbal Learning Test; Nonverbal Learning Test; Standard Progressive Matrices	NA	Sociodemographic; Health‐related behavioral; Clinical conditions	PaO_2_; PaCO_2_; SO_2_	7
	31	Kozora; 1999	United States	32	31	70.10	53.97	Diagnosis of COPD based on the criteria of the American Thoracic Society	Trails B; Digits forward; Digits backward; Verbal retention; Visual retention; Verbal pairs I & II	NA	NA	PaO_2_	7
	32	Krishnamurthy; 2019	India	42	42	51.52	76.00	Diagnosed stable COPD based on GOLD criteria	MMSE	NA	NA	SpO_2_	7
	33	Li; 2013	China	62	27	68.30	71.91	The diagnoses and classification of COPD were made according to the GOLD 2011 guidelines	MMSE	NA	NA	PaO_2_; PaCO_2_; SO_2_	7
	34	Özge; 2006	Turkey	54	24	63.92	80.77	Diagnosis of COPD according to GOLD criteria	MMSE	MMSE score < 24 indicated a generic cognitive dysfunction	NA	PaO_2_; PaCO_2_	6
	35	Pierobon; 2018	Italy	65	372	69.90	72.30	Diagnosed as stage II–IV according to GOLD criteria	Digit Span; TMT‐B; TMT‐A; Abstract Verbal Reasoning; Episodic Memory: IR/DR; Interference memory 10″/30″; Clock Drawing; Overlapping Pictures; Spontaneous/Copy Drawing; Ideative/Ideomotor Praxis; Phonemic fluency	NA	Sociodemographic	NA	7
	36	Salık; 2007	Turkey	32	26	66.25	55.17	According to GOLD‐2004 criteria	MMSE	NA	NA	PaO_2_; PaCO_2_; SO_2_	7
	37	Fekri; 2017	Iran	87	60	59.52	81.63	Patients were considered to have COPD if they had FEV1/FVC < 0.7; and if it remained < 0.7 15 min after the administration of two puffs of the salbutamol inhaler	MMSE	NA	NA	NA	7
	38	Thakur; 2010	United States	1202	302	58.26	41.88	According the GOLG criteria	MMSE	Score of 24 points out of the MMSE total possible 30 points to indicate cognitive impairment	Sociodemographic; Health‐related behavioral	NA	7
COPD	39	Xiao; 2022	Netherlands	612	3080	67.63	46.32	COPD1 (FEV1/FVC < 70% and FEV1 ≥ 80% predicte); COPD 2–4 (FEV1/FVC < 70% and FEV1 < 80% predicted)	Letter‐Digit Substitution Test; Stroop Test; Word learning test; Word Fluency test; Purdue Pegboard test	NA	NA	NA	8
	40	Villeneuve; 2012	Canada	45	50	67.90	37.90	Clinically stable moderate to severe COPD according to the GOLD classification; postbronchodilation FEV1/FVC < 80% of the predicted normal value andFEV1 to FVC ratio < 0.7	NA	Cognitive impairment was defined by (1) a subjective complaint (structured interview or CFQ ≥ 24 or ≥ 1 item scored ≥ 3); (2) objective decline in ≥ 2 cognitive test scores (≥ 1.5 SD below mean; adjusted for age/education); (3) preserved activities of daily living; and (4) absence of other medical or psychiatric explanations	NA	SO_2_	7
	41	Siraj; 2021	United States	64,397	243,420	65.80	52.56	Based on Read codes in consultation with a geriatrician, with ≥ 1 year of record prior to the COPD diagnosis	NA	Patients had a doctor recorded diagnosis of cognitive impairment	NA	NA	8

Abbreviations: ACRT, Auditory Recognition Reaction Time; AHI, apnea hypopnea index; ASRT, Auditory Simple Reaction Time; BKSCA, Brief Kingston Standardized Cognitive Assessment; CMS, Chronic Mountain Sickness; COPD, chronic obstructive pulmonary disease; DVBM, Delayed Verbal Memory; GOLD, Global initiative for Chronic Obstructive Lung Disease; HVLT, Hopkins Verbal Learning Test; IVBM, Immediate Verbal Memory; MCI, mild cognitive impairment; MDCST, Multi Domain Cognitive Screening Test; MMSE, Mini Mental State Examination; MoCA, Montreal Cognitive Assessment; NA, not explored in the present papers; ODI, oxygen desaturation index; OSA, obstructive sleep apnea; RSPM, Raven's Standard Progressive Matrices; TMT‐A, Trail‐Making Test part A; TMT‐B, Trail‐Making Test part B.

^a^ Confounding factors in statistical models were assigned to six domains. (1) Sociodemographic: age, gender, education, urbanization level, geographical area, race, marital status, perceived financial status, family income, field center, socioeconomic status. (2) Health‐related behavior: body mass index (BMI), physical activity, smoking status, alcohol intake, self‐rated health status, C‐reactive protein (CRP), total cholesterol, HDL cholesterol, low‐density lipoprotein, glomerular filtration rate (eGFR), serum iron, chronic pain. (3) Clinical conditions/comorbidities: cardiovascular disease, coronary heart disease (CAD), congestive heart failure, hypertension (HT), diabetes mellitus (DM), cerebrovascular disease, stroke, chronic obstructive pulmonary disease (COPD), chronic kidney disease (CKD), cancer, peptic ulcer, dementia, depression, anxiety, PHQ‐9 scores, Charlson's Index, thyroid stimulating hormone levels, inflammation levels, leukocytes, pulmonary disease, myocardial infarction, peripheral vascular disease, hemiplegia, sleep apnea. (4) Genetic factors: apolipoprotein E ε4 (APOE4). (5) Premorbid cognition and sleep status: sleep apnea. (6) Study characteristics: field center.

All included studies were non‐randomly sampled observational studies (with potential selection bias), but of overall high quality: 30 were rated as high quality and 11 as moderate quality. Detailed assessments are provided in Table S3.

### 3.2 Associations Between CMS, Anemia, OSA, COPD and Cognitive Impairment

Thirteen studies (332, 598 participants) evaluated the associations between these conditions and cognitive impairment, using healthy population prevalence as a reference (Table S4, Figures [Link], [Link], [Link], [Link], [Link]). Chronic high‐altitude exposure (OR = 6.892, 95% CI: 2.429–19.559, *p* < 0.001), OSA (OR = 3.879, 95% CI: 2.969–5.066, *p* < 0.001), and COPD (OR = 1.370, 95% CI: 1.327–1.414, *p* < 0.001) all showed significant associations with increased cognitive impairment risk. Anemia (OR = 1.382, 95% CI: 1.247–1.532, *p* < 0.001) was significantly associated with increased cognitive impairment risk in dichotomous outcome analysis. This contrasts with its nonsignificant effect on continuous cognitive function scores (Section 3.3.1), a discrepancy explored in depth in Section 4.2.1.

Random‐effects meta‐analysis of all 13 studies confirmed a significant overall association (OR = 2.407, 95% CI: 1.833–3.161, *p* < 0.001) but with high heterogeneity (*I* ^2^ = 90.7%, *p* < 0.001).

### 3.3 Associations Between CMS, Anemia, OSA, COPD and Cognitive Function

A three‐level multivariable random‐effects meta‐analysis (REML) integrated 171 effect sizes, covering 35 studies, 108 “study‐cognitive outcome” combinations, and 171 “study‐outcome‐assessment tool” subgroups. Results are as follows.

#### 3.3.1 CMS, Anemia, OSA, COPD and Global Cognitive Function

Treating disease type (CMS, Anemia, OSA, and COPD) as a moderator had significant effects on global cognitive function (QM [df = 4] = 31.4368, *p* < 0.0001). Compared with healthy controls, COPD (SMD = −0.5749, 95% CI: −0.8563 to −0.2935, *p* < 0.001), chronic high‐altitude exposure (SMD = −0.5577, 95% CI: −1.0037 to −0.1116, *p* = 0.0143), and OSA (SMD = −0.4796, 95% CI: −0.7944 to −0.1648, *p* = 0.0028) were associated with significant and moderate cognitive decline. Anemia exerted a small, nonsignificant effect on global cognitive function (SMD = −0.1504, 95% CI: −0.5734 to 0.2727, *p* = 0.4860) (Table S5). This null continuous outcome contrasts with the significant cognitive impairment risk (OR) in Section 3.2, a discrepancy potentially arising from population differences, assessment tool sensitivity, or disease severity stratification.

#### 3.3.2 Chronic Hypoxic Diseases and Cognitive Function Domains

**FIGURE 2:**
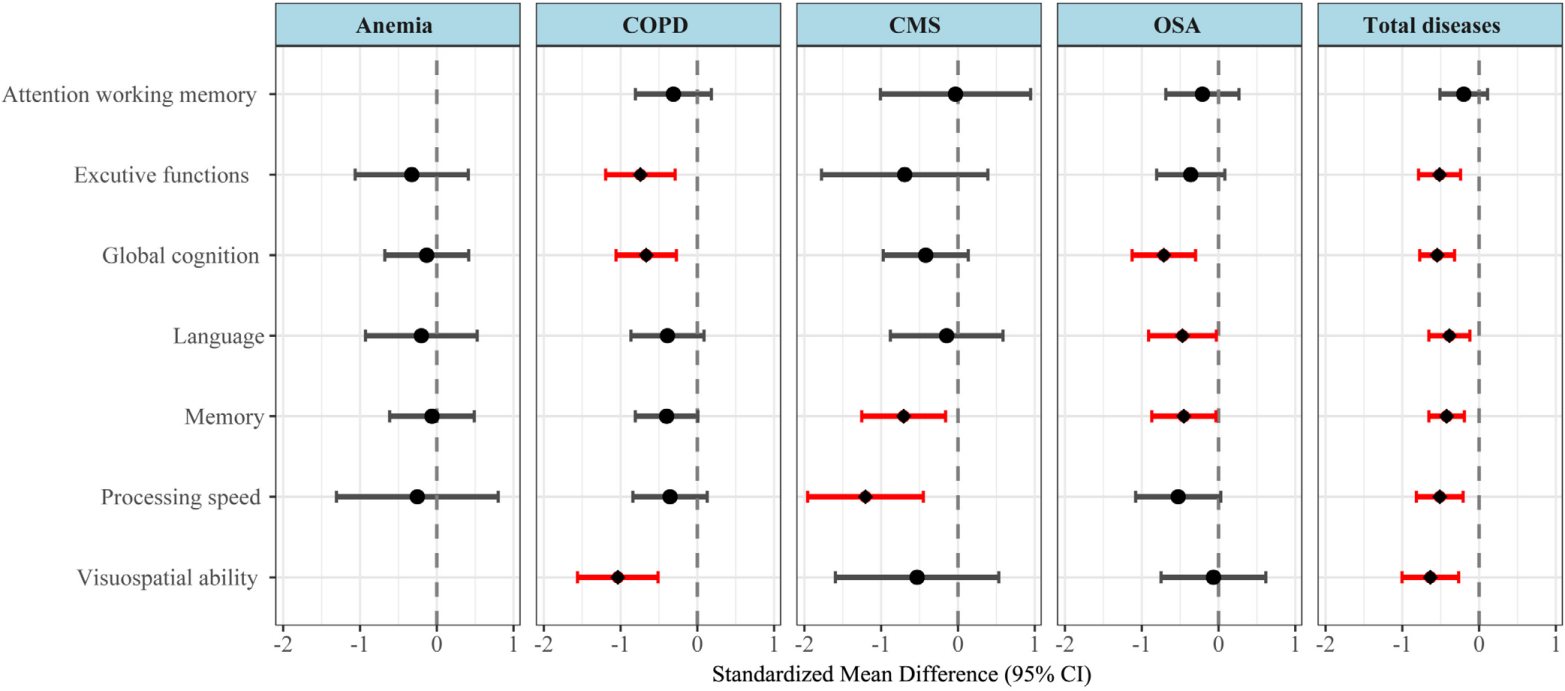
Comparative standardized mean differences of cognitive domains by disease group. Red lines indicate significant differences (*p* < 0.05); the absence of confidence intervals and point estimates indicates not applicable. CI, Confidence interval; CMS, Chronic Mountain Sickness; COPD, Chronic Obstructive Pulmonary Disease; OSA, Obstructive Sleep Apnoea; SMD, standardized mean differences.

Cognitive domain type significantly moderated the effects (QM [df = 7] = 36.0884, *p* < 0.0001). Integrating the pooled effects of all four chronic hypoxic diseases, significant moderate impairments were observed in multiple core cognitive domains, including global cognition, visuospatial ability, executive function, processing speed, memory, and language function. Specific statistical details (SMD values, 95% CIs and *p* values) are summarized in Figure 2 and Table S6.

#### 3.3.3 CMS, Anemia, OSA, COPD, and Cognitive Domains

The “disease type × cognitive domain” interaction significantly moderated effects (QM [df = 26] = 56.9115, *p* = 0.0004). The most notable disease‐cognitive domain pairings from the interaction analysis were as follows: Specific statistical details (SMD values, 95% CIs and *p* values) are summarized in Figure 2 and Table S6, and the interaction patterns are as follows: Anemia showed no significant associations with any cognitive domain (all *p* > 0.05); CMS caused significant moderate‐to‐large impairments mainly in processing speed and memory; OSA was linked to significant impairments in language and memory, with a particularly prominent impairment in global cognition; COPD induced significant moderate‐to‐large impairments in visuospatial ability and global cognition, plus moderate impairment in executive function.

#### 3.3.4 Subgroup Analyses

Subgroup analyses (age, sample size, region, and hypoxia indicators) explored heterogeneity sources, with significant “subgroup × cognitive outcome” interactions (all QM *p* < 0.0001) (Table 2).

**TABLE 2:** Subgroup analyses of standardized mean differences in cognitive domains stratified by age, sample size, continent, and hypoxia indicators.

	*N*	Executive functions SMD (95% CIs)^a^	Memory SMD (95% CIs)^a^	Processing speed SMD (95% CIs)^a^	Language SMD (95% CIs)^a^	Attention working memory SMD (95% CIs)^a^	Visuospatial ability SMD (95% CIs)^a^	Global cognition SMD (95% CIs)^a^
Age								
< 65 years	119	−0.4776 (−0.8158, −0.1393)**	−0.4859 (−0.7646, −0.2071)***	−0.6724 (−1.0454, −0.2994)***	−0.3989 (−0.7337, −0.0641)*	−0.1772 (−0.5562, 0.2017)	−1.1287 (−1.7006, −0.5569)***	−0.5817 (−0.8355, −0.3279)***
≥ 65 years	52	−0.5269 (−1.0040, −0.0498)*	−0.2819 (−0.7021, 0.1382)	−0.1904 (−0.7283, 0.3474)	−0.3214 (−0.7764, 0.1336)	−0.2574 (−0.8057, 0.2909)	−0.2726 (−0.7906, 0.2455)	−0.4811 (−1.0509, 0.0886)
Sample size								
Small sample (*n* < 100)	61	−0.7472 (−1.1797, −0.3147)***	−0.6672 (−1.0809, −0.2536)**	−1.2366 (−1.8281, −0.6451)***	−0.7571 (−1.2483, −0.2658)**	−0.2810 (−0.7714, 0.2094)	0.0416 (−0.8414, 0.9246)	−0.8548 (−1.1868, −0.5528)***
Large sample (*n* ≥ 100)	110	−0.3609 (−0.6780, −0.0437)*	−0.2691 (−0.5192, −0.0190)*	−0.2540 (−0.5834, 0.0755)	−0.1705 (−0.4618, 0.1209)	−0.1384 (−0.5036, 0.2268)	−0.6419 (−1.0269, −0.2569)**	−0.3127 (−0.5850, −0.0404)*
Continent								
Asia	55	−0.2869 (−0.8530, 0.2792)	−0.5216 (−0.8777, −0.16568)**	−0.9015 (−1.4566, −0.3463)**	−0.22599 (−0.7037, 0.2519)	−0.0843 (−0.7636, 0.5950)	−0.4498 (−1.4651, 0.5655)	−0.5161 (−0.8083, −0.2238)***
Europe	57	−0.7741 (−1.2127, −0.3355)***	−0.3446 (−0.7108, 0.0216)	−0.3101 (−0.7750, 0.1549)	−0.4252 (−0.8485, −0.0019)*	−0.3475 (−0.8247, 0.1297)	−0.7920 (−1.2499, −0.3342)***	−0.4395 (−0.9233, 0.0443)
Africa	6	−2.4203 (−4.1653, −0.6753)**	NA	NA	NA	−0.6363 (−2.1978, 0.9253)	NA	−1.6969 (−2.5142, −0.8796)***
America	41	−0.1746 (−0.6528, 0.3035)	−0.0529 (−0.5247, 0.4189)	−0.1018 (−0.7822, 0.5787)	−0.1267 (−0.6339, 0.3804)	0.1842 (−0.3944, 0.7629)	−0.0288 (−0.9480, 0.8904)	−0.4522 (−1.0610, 0.1566)
Oceania	12	−0.1813 (−1.2915, 0.9289)	−1.0333 (−2.1194, 0.0527)	−0.8556 (−1.9406, 0.2293)	−0.8403 (−1.9697, 0.2890)	−0.7693 (−2.0237, 0.4851)	NA	−0.0330 (−1.1929, 1.1270)
Hypoxia indicators								
With hypoxia indicators	98	−0.6316 (−1.0051, −0.2581)***	−0.5668 (−0.8899, −0.2437)***	−0.8074 (−1.2903, −0.3244)**	−0.5530 (−0.9221, −0.1838)**	−0.2956 (−0.6760, 0.0848)	−1.0962 (−1.6929, −0.4994)***	−0.7405 (−1.0526, −0.4284)***
Without hypoxia indicators	73	−0.3914 (−0.7869, 0.0042)	−0.2663 (−0.5831, 0.0506)	−0.2778 (−0.6752, 0.1196)	−0.1846 (−0.5663, 0.1971)	−0.1389 (−0.6882, 0.4103)	−0.2969 (−0.7800, 0.1861)	−0.3424 (−0.6607, −0.0240)*

*Note:* Significance levels: *p < 0.05; **p < 0.01; ***p < 0.001.

Abbreviations: CIs, Confidence interval; *N*, number of effect sizes; NA, not applicable; SMD, standardized mean differences.

^a^ The random‐effects model was utilized.

Age: Participants < 65 years showed significant moderate‐to‐large impairments in language (SMD = −0.3989, *p* = 0.0195), executive function (SMD = −0.4776, *p* = 0.0057), memory (SMD = −0.4859, *p* = 0.0006), global cognition (SMD = −0.5817, *p* < 0.0001), processing speed (SMD = −0.6724, *p* = 0.0004), and visuospatial ability (SMD = −1.1287, *p* = 0.0001). Those ≥ 65 years only had significant executive function impairment (SMD = −0.5269, *p* = 0.0304).

Sample size: Small‐sample studies (*n* < 100) showed more severe impairments in memory (SMD = −0.6672, *p* = 0.0016), executive function (SMD = −0.7472, *p* = 0.0007), language (SMD = −0.7571, *p* = 0.0025), global cognition (SMD = −0.8548, *p* < 0.0001), and processing speed (SMD = −1.2366, *p* < 0.0001). Large‐sample studies (*n* ≥ 100) had partial significant impairments in memory (SMD = −0.2691, *p* = 0.0350), global cognition (SMD = −0.3127, *p* = 0.0244), executive function (SMD = −0.3609, *p* = 0.0257), and visuospatial ability (SMD = −0.6419, *p* = 0.0011).

Region: African populations had the most severe executive function (SMD = −2.4203, *p* = 0.0066) and global cognition (SMD = −1.6969, *p* < 0.0001) impairments. Asian populations showed deficits in global cognition (SMD = −0.5161, *p* = 0.0005), memory (SMD = −0.5216, *p* = 0.0041), and processing speed (SMD = −0.9015, *p* = 0.0015). European populations had impairments in language (SMD = −0.4252, *p* = 0.0490), executive function (SMD = −0.7741, *p* = 0.0005), and visuospatial ability (SMD = −0.7920, *p* = 0.0007). No significant associations were found in the Americas or Oceania.

Hypoxia indicators: Groups with hypoxia indicators showed moderate‐to‐large impairments in language (SMD = −0.5530, *p* = 0.0033), memory (SMD = −0.5668, *p* = 0.0006), global cognition (SMD = −0.7405, *p* < 0.0001), executive function (SMD = −0.6316, *p* = 0.0009), processing speed (SMD = −0.8074, *p* = 0.0011), and visuospatial ability (SMD = −1.0962, *p* = 0.0003). Those without showed only marginally significant global cognition impairment (SMD = −0.3424, *p* = 0.0351).

### 3.4 Publication Bias and Sensitivity Analysis

In the disease‐cognitive impairment correlation analysis, the funnel plot (Figure S6) and Egger's test explicitly confirmed significant publication bias (*P* = 0.017). After trim‐and‐fill correction, the pooled OR for this association dropped from 2.407 (95% CI: 1.833–3.161, *p* < 0.001) to a nonsignificant value (adjusted OR = 1.504, 95% CI = 0.910–2.482), indicating that the observed association was not robust to adjustment for publication bias.

In the study on the association between diseases and cognitive function, publication bias analysis showed: visual assessment of the funnel plot revealed that most study points were concentrated within the pseudo 95% confidence interval, but the density of study points in the region with low standard error on the left side (negative effect size direction) was slightly lower than that on the right side, showing a slight asymmetry (Figure S7); Egger's mixed‐effects meta‐regression test indicated significant publication bias (intercept = 0.0656, 95% CI: −0.0533–0.1846, *z* = −8.7021, *p* < 0.0001); further correction using the trim‐and‐fill method detected no studies requiring imputation, and the corrected pooled effect size was consistent with the original effect size (original effect size = −0.39, 95% CI: −0.4713 to −0.3088; corrected effect size = −0.39, 95% CI: −0.4713 to −0.3088).

Sensitivity analysis showed that after excluding any single study in the correlation analysis between diseases and cognitive impairment, the direction of the overall estimate remained unchanged, and there was no change in heterogeneity. Subgroup analysis by disease revealed that heterogeneity was reduced in all disease subgroups except for chronic high‐altitude exposure (Table S4).

In the study on the association between diseases and cognitive function, comparison of the three‐level model and two‐level model using the LRT showed that the three‐level model had a significantly better goodness of fit (all *p*‐values < 0.0001). Analyses of the two models showed that although there were differences in effect sizes, the results were generally consistent (Tables S5–S8).

## 4 Discussion

This is the first meta‐analysis to systematically explore associations and mechanisms linking four chronic hypoxic diseases—CMS, anemia, OSA, and COPD—and cognitive impairment, with each exhibiting specificity in damaging cognitive subdomains. Notably, while anemia shows an epidemiological link to elevated cognitive impairment risk, it has no statistically significant impact on global or domain‐specific cognitive scores. It is critical to highlight that this study has a methodological limitation: publication bias was detected in the cognitive impairment risk analysis; trim‐and‐fill correction nullified the statistical significance of the pooled OR. This finding suggests that the original effect size may have been inflated by selective reporting, and the conclusion that chronic hypoxic diseases significantly increase the risk of cognitive impairment should therefore be interpreted with caution.

Hypoxia serves as the core mediator of these associations, involving shared pathways such as energy metabolism dysfunction, oxidative stress, and neuroinflammation, alongside unique disease‐specific pathogenic processes modulated by genetic polymorphisms and physiological adaptation. These findings provide evidence for clinical cognitive screening in patients with chronic hypoxic diseases, while also identifying key limitations of the current study. Future research should adopt standardized designs to further verify causality and explore precise preventive and therapeutic strategies.

### 4.1 Mechanisms: Pathway Analysis From Hypoxia to Cognitive Impairment (Table 3)

**TABLE 3:** Mechanisms underlying the association between chronic hypoxic diseases (CMS, Anemia, OSA, and COPD) and cognitive impairment.

Disease	Core differences	Common mechanisms	Unique mechanisms	Adaptive mechanisms	Genetic factors	Cognitive impairment				
						Memory	Language	Executive function	Attention	Visuospatial ability
CMS	Centered on “long‐term hypoxia → excessive increase in red blood cells → high blood viscosity,” without exogenous nutrient deficiency or primary abnormalities in sleep/lung function	Chronic/intermittent hypoxia: activation of HIF‐1α, mitochondrial dysfunction, and insufficient neuronal energy Oxidative stress and inflammation: accumulation of ROS, release of IL‐1β/TNF‐α, activation of microglia, and damage to the blood–brain barrier Abnormal cerebral blood flow: insufficient or fluctuating cerebral perfusion, and decreased cerebrovascular reactivity Neurostructural damage: reduced hippocampal synaptic plasticity and white matter demyelination	Increased hematocrit → stasis of cerebral microcirculation Long‐term hypoxia → gray matter atrophy Decreased dopa decarboxylase activity → reduced dopamine synthesis	Negative feedback regulation of the HIF pathway Cerebral microvascular neogenesis Activation of the antioxidant system	▲EPAS1 gene (HIF‐2α) ▲EGLN1 gene (PHD2 encoding gene) ▲ APOE ε2 allele	Decline in episodic memory	Naming difficulties	Difficulty in task switching	Distraction of selective attention	Abnormal depth perception
Anemia	Centered on “insufficiency of oxygen carriers (hemoglobin/red blood cells),” accompanied by direct neurotoxicity of nutrients such as iron/folate, and unrelated to oxygen partial pressure		Decreased iron → impairment of myelin synthesis Decreased red blood cells → disorder of blood flow regulation Increased homocysteine → abnormal neuronal DNA methylation	Circulatory compensation Iron metabolism regulation Enhanced glycolysis	▲TMPRSS6 gene ▲HFE gene (C282Y mutation) ▲ BDNF gene (Val66Met)	Impaired short‐term and delayed recall	Slow lexical retrieval	Decreased decision‐making ability	Difficulty sustaining attention	Impaired spatial navigation
OSA	Centered on “intermittent hypoxia + sleep fragmentation,” with periodic stress amplifying damage, and sleep structure disruption independently causing pathogenesis		Hypoxia‐reoxygenation cycle → amplified inflammation REM sleep deprivation Repeated sympathetic nerve activation → microvascular microinfarction	Sleep structure compensation Adaptive downregulation of the sympathetic nervous system Activation of mitophagy	▲ APOE ε2 allele ▲ TNF‐α gene (−308G/A) ▲ PER3 gene (clock gene)	Decline in procedural memory and working memory	Decreased speed of language comprehension	Impaired working memory	Great fluctuations in attention	Decline in visuospatial integration and graph recognition ability
COPD	Centered on “pulmonary dysfunction → systemic inflammation + gut‐lung‐brain axis disorder,” with chronic hypercapnia exacerbating neural inhibition		Imbalance of the gut‐lung‐brain axis → decreased 5‐hydroxytryptamine Chronic hypercapnia → reduced neuronal electrical activity Respiratory muscle fatigue → decreased cerebral oxygen	Pulmonary vascular remodeling Gut‐lung‐brain axis compensation Increased BDNF secretion	▲ACE gene (I/D polymorphism) ▲IL‐6 gene (−174G/C) ▲ CHRNA3 gene (nicotinic receptor gene)	Decline in episodic memory and associative memory	Deterioration in language organization ability	Reduction in problem‐solving ability	Impairment in attention distribution ability	Disorders in spatial composition and sense of direction

*Note:* A key transcription factor regulating cellular responses to hypoxia; IL‐1β, Interleukin‐1β, a pro‐inflammatory cytokine; TNF‐α, Tumor Necrosis Factor‐α, a pro‐inflammatory cytokine; ROS, Reactive Oxygen Species, critical mediators of oxidative stress; PHD2, Prolyl Hydroxylase Domain 2, an enzyme that regulates HIF degradation; APOE, Apolipoprotein E, involved in lipid metabolism and associated with cognitive function; BDNF, Brain‐Derived Neurotrophic Factor, promoting neuronal survival and differentiation; EPAS1, Endothelial PAS Domain Protein 1, encoding HIF‐2α (a hypoxia‐responsive transcription factor); EGLN1, Egl Nine Homolog 1, encoding PHD2 (regulates HIF degradation); TMPRSS6, Transmembrane Protease, Serine 6, involved in iron metabolism regulation; HFE, Hemochromatosis Gene, associated with iron metabolism disorders; PER3, Period Circadian Regulator 3, a clock gene involved in circadian rhythm regulation; Val66Met, Val66Met polymorphism in the BDNF gene, influencing BDNF function and secretion; −308G/A, −308G/A promoter polymorphism in the TNF‐α gene, regulating TNF‐α expression; I/D polymorphism, Insertion/Deletion polymorphism in the ACE gene, affecting ACE activity; −174G/C, −174G/C promoter polymorphism in the IL‐6 gene, regulating IL‐6 expression; CHRNA3, Cholinergic Receptor Nicotinic Alpha 3, involved in neuronal signaling; REM, Rapid Eye Movement sleep, a stage of sleep associated with dreaming; ▲, indicates a significant association between the gene and the corresponding phenotype; →, denotes a causal or regulatory relationship (e.g., “A → B” means A regulates or causes B).

Abbreviations: CMS, Chronic Mountain Sickness; COPD, Chronic Obstructive Pulmonary Disease; OSA, Obstructive Sleep Apnoea; HIF‐1α, Hypoxia‐Inducible Factor‐1α.

#### 4.1.1 Shared Pathogenic Mechanisms

Despite distinct etiologies, all four diseases converge on chronic hypoxia as a core initiating factor, driving cognitive impairment via shared pathways.

##### 4.1.1.1 Energy Metabolism Disruption

Hypoxia activates HIF‐1α, inhibiting mitochondrial oxidative phosphorylation [61], reducing neuronal energy supply—particularly in cognition‐critical regions (hippocampus, prefrontal cortex).

##### 4.1.1.2 Oxidative Stress and Neuroinflammation

Reactive oxygen species (ROS) accumulation induces lipid/protein oxidation (e.g., abnormal tau phosphorylation), while microglial activation releases pro‐inflammatory cytokines (IL‐6, TNF‐α) [62], damaging the blood–brain barrier, neurons, and hippocampal/white matter integrity.

##### 4.1.1.3 Cerebrovascular Dysregulation

Includes cerebral hypoperfusion (COPD, anemia), cerebral blood flow fluctuations (OSA intermittent hypoxia), and reduced cerebrovascular reactivity (CMS). These impair neuronal glucose uptake, exacerbating damage.

##### 4.1.1.4 Neural Structural and Functional Damage

Impaired synaptic plasticity (anemia, OSA, COPD) and white matter demyelination (e.g., COPD inflammation, OSA hypoxia‐reoxygenation stress) disrupt signaling, causing deficits in executive function, memory, and other domains [63, 64].

#### 4.1.2 Disease‐Specific Pathogenic Mechanisms

Beyond shared hypoxia‐driven pathways, unique pathological features further exacerbate cognitive decline.

##### 4.1.2.1 CMS

Sustained HIF‐1α overexpression triggers excessive erythropoietin (EPO) secretion [65, 66], leading to erythrocytosis, hyperviscosity, and impaired cerebral microcirculation. Chronic hypoxia also accumulates mitochondrial DNA (mtDNA) mutations [67, 68], depletes neuronal energy, and reduces dopamine synthesis (via inhibited dopa decarboxylase), disrupting signaling.

##### 4.1.2.2 Anemia

Iron‐deficiency impairs myelin synthesis, damaging white matter [69]; folate/B12 deficiency elevates homocysteine, inducing abnormal neuronal DNA methylation and impairing neurogenesis/repair [70].

##### 4.1.2.3 OSA

Nocturnal hypoxia‐reoxygenation cycles activate the NLRP3 inflammasome, while sleep fragmentation (especially loss of deep sleep) impairs memory consolidation [71, 72]. Recurrent sympathetic activation induces cerebral small vessel disease (microinfarcts/microbleeds), directly damaging cognition‐related regions.

##### 4.1.2.4 COPD

Gut‐lung‐brain axis dysregulation disrupts gut microbiota, reducing serotonin synthesis [73]; chronic hypercapnia lowers cerebrospinal fluid pH, inhibiting neuronal activity; respiratory muscle fatigue exacerbates cerebral oxygen imbalance—collectively impairing cognition.

#### 4.1.3 Genetic Specificity and Physiological Adaptation

The severity of cognitive impairment is regulated by individual genetic background and compensatory capacity; population/geographic differences in gene polymorphisms are the core mechanism underlying effect size heterogeneity in subgroup analyses.

##### 4.1.3.1 Gene Polymorphism

In Chronic Mountain Sickness (CMS), variations in Endothelial PAS Domain Protein 1 (EPAS1, e.g., rs1867785) and Egl‐9 Family Hypoxia‐Inducible Factor 1 (EGLN1, e.g., rs12097901)—key HIF pathway regulators—are core loci for high‐altitude hypoxia adaptation [74, 75, 76]. These adaptive variations are highly prevalent in long‐term high‐altitude populations (e.g., Tibetans, Andean natives), inhibiting pathological polycythemia (EPAS1) and alleviating hypoxic neuronal injury (EGLN1), leading to significantly lower CMS‐related cognitive impairment effect sizes than in low‐altitude and unadapted immigrant populations (subgroup effect size difference ≥ 0.3). In contrast, CMS patients migrating from plains to high altitudes have < 20% carrier rate of these protective variations, resulting in weaker hypoxia adaptation and higher effect sizes.

Apolipoprotein E (APOE) ε2 protects hippocampal neurons by reducing β‐amyloid peptide (Aβ) deposition [76, 77, 78], with carrier rates varying significantly by region/ethnicity (15%–20% in East Asians, 10%–15% in Europeans, 5%–10% in Africans) [79]. This aligns with subgroup analyses showing lower Obstructive Sleep Apnea (OSA)/Chronic Obstructive Pulmonary Disease (COPD)‐related cognitive impairment effect sizes in East Asians, attributed to higher APOE ε2 carrier rates enhancing neuroprotection.

In anemia, Transmembrane Protease, Serine 6 (TMPRSS6, e.g., rs855791) variation alleviates cognitive injury by enhancing iron absorption [80]. Its carrier rate is 30%–40% in iron‐deficient regions (e.g., South Asia, sub‐Saharan Africa) vs. < 10% in iron‐sufficient regions (e.g., North America) [81], compensating for iron‐deficiency and reducing anemia‐related cognitive impairment effect sizes in iron‐deficient regions.

In COPD, Angiotensin‐Converting Enzyme 2 (ACE2) and Cholinergic Receptor Nicotinic Alpha 3 Subunit (CHRNA3) polymorphisms correlate with cognitive impairment effect sizes. Southeast Asians have higher protective ACE II genotype carrier rates than Europeans [82], while European smokers have higher CHRNA3 risk variation (rs1051730) rates than Asian smokers [83], explaining lower COPD‐related cognitive impairment in Southeast Asians and higher in European smokers.

##### 4.1.3.2 Physiological Adaptation

CMS populations inhibit polycythemia via HIF pathway feedback [84, 85], compensate for insufficient cerebral perfusion through microvascular neogenesis, and reduce reactive oxygen species injury via antioxidant activation [86]. Anemia patients offset oxygen deficiency through circulatory compensation, iron metabolism regulation, and enhanced glycolysis [87]. OSA patients alleviate memory impairment by adjusting sleep structure [88] and reduce mitochondrial DNA mutations via mitophagy [89, 90]. COPD patients upregulate Brain‐Derived Neurotrophic Factor (BDNF) secretion to promote prefrontal synaptic regeneration and retain partial executive function [91, 92].

Population‐specific gene polymorphism distributions, combined with physiological adaptive responses, partially antagonize hypoxic neuronal injury, contributing significantly to the heterogeneity of cognitive impairment effect sizes across populations in subgroup analyses.

### 4.2 Consensus, Controversies, and Significance of the Study

#### 4.2.1 Consensus

This study confirms that CMS, anemia, OSA, and COPD are associated with elevated risks of cognitive impairment, aligning with recent findings [93, 94, 95, 96].

In terms of cognitive subdomains, CMS correlates with multi‐domain deficits in attention, memory, and executive function—linked to cerebral microcirculatory dysfunction and neural structural damage (e.g., reduced hippocampal synaptic plasticity) from chronic high‐altitude hypoxia, consistent with high‐altitude research [97]. For anemia, our analysis found no significant negative associations with any cognitive subdomain (e.g., executive function, global cognition, memory), a stark contrast to the significant anemia‐related cognitive impairment risk in dichotomous analyses (OR = 1.382, 95% CI: 1.247–1.532, *p* < 0.001). Three key factors explain this apparent inconsistency: (1) Dichotomous analyses prioritized severe, comorbid anemia cases meeting overt cognitive impairment criteria, while continuous analyses included mild, community‐dwelling patients [51]; (2) Broad dichotomous thresholds (e.g., MMSE < 24) may overclassify cognitive impairment, whereas domain‐specific continuous tests may lack sufficient sensitivity to mild anemia‐induced subtle deficits [13, 50]; (3) Physiological compensation (e.g., elevated cerebral blood flow, upregulated glycolysis) masks minor cognitive changes in mild anemia but is ineffective in severe cases [98]. This finding of nonsignificant associations in continuous analyses is inconsistent with the conclusions of cross‐sectional studies demonstrating that iron‐deficiency anemia impairs memory function [99, 100], but aligns with baseline data from the Brazilian cohort and a study of Chinese adults over 65, which found no significant anemia‐cognitive function links (global or subdomain) after covariate adjustment [51, 101]. OSA exhibits a clear dose effect: memory and language function deteriorate with increasing AHI [18, 102]. Notably, structural and functional abnormalities in left‐hemisphere regions further validate the view that “intermittent hypoxia + sleep fragmentation impairs cognitive function by disrupting the left‐hemisphere dominance effect” [103, 104]. Consistent with other research [105, 106, 107], COPD patients show significant impairments in executive function, attention, memory, and visuospatial ability. Mendelian randomization [108] and biomedical studies [109] link COPD‐related systemic inflammation and oxidative stress to cognitive decline.

Together with existing literature, our findings confirm chronic hypoxia as a key driver of cognitive impairment. Consensus exists that OSA and COPD, as critical risk factors for cognitive decline, act via hypoxia, inflammation, and related pathways. Additionally, more severe cognitive impairment in individuals with positive hypoxia indicators strengthens support for a causal “hypoxia‐cognitive impairment” axis.

#### 4.2.2 Controversies

Subgroup analyses revealed variability by sample size, region, and age:

Sample size/region: Smaller studies showed higher homogeneity but greater selection bias; larger studies had higher heterogeneity. Regional differences likely stem from diagnostic criteria, assessment tool cultural adaptability, and genetic background.

Age: < 65‐year‐olds exhibited multi‐domain impairments (language, executive function, memory, processing speed, and visuospatial ability), while ≥ 65‐year‐olds only had executive function deficits. This likely reflects interactions between hypoxia severity, cerebral reserve, and comorbidities: younger individuals with intact reserve cannot fully compensate for acute/high‐intensity hypoxia, damaging “non‐aging networks” [110, 111]. In contrast, older individuals, with reduced neurovascular reactivity and prefrontal aging, show selective executive function vulnerability, exacerbated by chronic persistent hypoxia from predominant COPD/anemia in this group—consistent with hypoxic elderly COPD patients [112]. Our post hoc analysis revealed a striking difference in disease composition between age groups: CMS/OSA (characterized by acute intermittent hypoxia) constituted the majority (55.6%) of studies in the < 65‐year‐old group, whereas COPD/anemia (characterized by chronic persistent hypoxia) accounted for 91.7% of the studies in the ≥ 65‐year‐old group. This suggests that the pattern of cognitive impairment may be driven not only by age but also by the distinct hypoxic profiles of the prevalent diseases in each age stratum.

#### 4.2.3 Significance

This study fills critical gaps by: being the first to systematically compare cognitive impacts across the four diseases, clarifying association strengths and subdomain specificity. Identifying Asian/African populations and those with positive hypoxia indicators as high‐risk groups, guiding targeted interventions. Addressing anemia‐related controversies with large‐sample evidence, informing future research. Emphasizing the clinical necessity of cognitive screening in COPD and OSA patients, with direct implications for practice.

### 4.3 Heterogeneity Analysis: Methodological Limitations and Biological Interpretations

#### 4.3.1 Severe Publication Bias in Cognitive Impairment Risk Analysis

A critical limitation of this study is significant publication bias in the meta‐analysis of the association between chronic hypoxic diseases and cognitive impairment risk (13 studies included). Egger's test confirmed significant publication bias (*p* = 0.017), and the pooled odds ratio (OR) for the overall association lost statistical significance after trim‐and‐fill correction, which directly weakens the reliability of the conclusion that chronic hypoxic diseases increase cognitive impairment risk.

This bias is mainly attributed to two factors: first, unpublished negative results from small‐sample studies—many small‐sample clinical studies failed to detect a significant association, and such negative/null results are often unsubmitted or rejected, leading to overrepresentation of positive findings; second, the publication bias toward positive results—journals tend to prioritize publishing statistically significant positive outcomes, while neglecting negative results, which further exacerbates funnel plot asymmetry and overestimation of effect sizes. Additionally, heterogeneity among included studies (e.g., study population, diagnostic criteria for cognitive impairment, follow‐up duration) may increase susceptibility to publication bias.

This severe publication bias significantly limits the certainty of the conclusion regarding the association between chronic hypoxic diseases and cognitive impairment risk: current data cannot accurately determine the true association, and the actual effect size may be lower than the original pooled OR (2.407). To verify the robustness of the conclusion, additional sensitivity analyses were performed by excluding small‐sample studies (*n* < 100) and moderate‐to‐low methodological quality (Newcastle‐Ottawa Scale score ≤ 6). The results showed a further reduced pooled OR (2.320, 95% CI: 1.755–3.068, *p* < 0 0.001) with persistent high heterogeneity (*I* ^2^ = 91.9%, *p* < 0.001), suggesting that the original effect size was overestimated due to publication bias and small‐sample interference. The true association between chronic hypoxic diseases and cognitive impairment risk requires verification by more large‐sample, high‐quality prospective studies.

#### 4.3.2 Sources of Heterogeneity and Publication Bias

The heterogeneity of results mainly stems from inconsistencies in three aspects: sociodemographic factors (age, sex, and region) and methodology. Specifically: Diverse disease diagnostic criteria: For OSA, the apnea‐hypopnea index (AHI) cutoff values vary, ranging from ≥ 5 to ≥ 30 events/h [52, 57, 58]; for COPD, different versions and stages of the GOLD criteria are referenced [15, 29]; for CMS, definitions of altitude and exposure duration are inconsistent [34, 45, 48]; and subtypes of anemia are not unified. These inconsistencies lead to differences in disease severity and hypoxic conditions among included patients.

Varied cognitive assessment tools: Studies on OSA use tools such as MMSE and MoCA, while studies on COPD employ tools like MMSE and TMT. Differences in the sensitivity and targeted cognitive domains of these tools contribute to result variations.

Nonuniform criteria for judging cognitive impairment: Criteria range from no clear threshold to MMSE < 24 points, MoCA < 26 points, or international MCI standards, further exacerbating heterogeneity.

Egger's test for the association between diseases and cognitive function suggested potential publication bias, but the trim‐and‐fill method identified no studies requiring imputation. This discrepancy may be related to methodological principles: Egger's test is more sensitive to minor asymmetries in the funnel plot, and may amplify random errors in small‐sample studies, especially when the number of included studies is small or effect sizes are large [113]; in contrast, the trim‐and‐fill method relies more on the “symmetric missing pattern” of the funnel plot. If asymmetry is not caused by “unpublished negative‐result studies” (e.g., due to heterogeneity), no imputation is needed [114]. Additionally, the pooled effect size did not substantially change after correction, suggesting that even if potential publication bias exists, its impact on the core conclusions of this study may be limited. However, it should be noted that when the number of included studies is small, the stability of publication bias detection methods decreases, and the above results require cautious interpretation [115].

#### 4.3.3 Biological Heterogeneity Mechanisms

Genetic polymorphisms regulating hypoxia tolerance, inflammation, and metabolism drive biological heterogeneity by modulating neuroprotection, repair, and compensatory capacity—shaping differential cognitive responses to the four diseases.

### 4.4 Clinical Implications: Translating Evidence Into Practice

#### 4.4.1 Optimization of Routine Management

Given the significant publication bias and internal data contradictions identified in this study, there is currently insufficient evidence to support the routine integration of chronic hypoxic diseases into cognitive impairment screening protocols. For patients with Chronic Obstructive Pulmonary Disease (COPD)/Obstructive Sleep Apnea (OSA), especially those with positive hypoxia indicators or of Asian/African descent, consideration may be given to cognitive function assessment using the Montreal Cognitive Assessment (MoCA), but the benefits of its routine application require further verification by high‐quality future studies. Alleviating hypoxia (via Continuous Positive Airway Pressure [CPAP] for OSA and pulmonary function optimization for COPD) may help slow cognitive decline, and this intervention effect needs confirmation by larger‐sample prospective studies.

#### 4.4.2 Individualized Interventions

Interventions targeting disease‐specific mechanisms are still in the exploratory stage: OSA management should address both hypoxia and sleep architecture improvement, and its protective effect on cognitive function requires further verification; COPD interventions may focus on gut‐lung‐brain axis regulation, whose clinical effectiveness awaits clarification by future research. Although the association between anemia and cognitive impairment is not statistically significant, age/subtype‐specific assessments are still needed to avoid missing potential risks. Monitoring hypoxia indicators as prognostic markers to preliminarily explore the establishment of a “screening‐intervention‐monitoring” loop requires high‐quality studies to further confirm its effect on improving patient outcomes.

### 4.5 Future Research Directions

#### 4.5.1 Mechanistic Exploration

Elucidate molecular/neural circuit pathways underlying hypoxia‐induced domain‐specific deficits (e.g., OSA‐related sleep fragmentation + hypoxia impacts on hippocampal memory circuits). Explore population differences, focusing on genetic polymorphism‐environment interactions in Asian/African populations.

#### 4.5.2 Methodological Improvements

Standardize cognitive assessment (uniform tools/cutoffs) to reduce heterogeneity. Conduct prospective cohort studies on anemia (stratified by subtype/duration) with long‐term hypoxia monitoring. Validate causality via interventional studies (e.g., hypoxia correction, anti‐inflammatory therapies) to refine clinical translation.

## 5 Conclusion

Hypoxia is the core shared mechanism linking CMS, anemia, OSA, and COPD—the four chronic hypoxic diseases explored in this study—to cognitive impairment, with each disease exhibiting specific damage to cognitive subdomains. As the first meta‐analysis to systematically explore this association, our findings reveal critical limitations: while anemia shows an epidemiological association with increased cognitive impairment risk, it exhibits no statistically significant difference in global or domain‐specific cognitive scores; notably, publication bias in the cognitive impairment risk analysis and subsequent trim‐and‐fill correction that nullified the statistical significance of the pooled odds ratio significantly weakens the reliability of our core conclusion, indicating varying certainty of evidence across diseases and outcomes. Beyond the core hypoxic mechanism, shared pathogenic pathways (energy metabolism dysfunction, oxidative stress, neuroinflammation) and unique disease‐specific processes (modulated by genetic polymorphisms and physiological adaptation) underlie this association. These findings support clinical cognitive screening and targeted interventions for patients with chronic hypoxic diseases based on current evidence. Future research should adopt standardized, longitudinal study designs to verify causality and explore precise preventive and therapeutic strategies, thereby strengthening the certainty of evidence for this association.

## Author Contributions

Haishi Fei, Guirong Cheng, and Yan Zeng contributed equally to this work and should be considered co‐first authors. Conceptualization: Shengzhong Yi and Guirong Cheng. Data curation: Haishi Fei and Zhichao He. Formal analysis: Haishi Fei and Feibo Zhao. Funding acquisition: Yan Zeng. Supervision: Shengzhong Yi. Writing – original draft: Haishi Fei and Guirong Cheng. Writing – review and editing: All authors.

## Funding

This study was supported by the Brain Science and Brain–like Intelligence Technology—National Science and Technology Major Project (2022ZD0211600).

## Ethics Statement

Ethical approval was not required for this study because it is a systematic review and meta‐analysis based on previously published data. The review protocol was registered in PROSPERO (CRD420251106030).

## Conflicts of Interest

The authors declare no conflicts of interest.

## Supporting information


**Appendix S1:** PRISMA 2020 main checklist.

**Figure S1:** Forest plot of associations between CMS and cognitive impairment: findings from a meta‐analysis. CIs, Confidence interval; CMS, Chronic Mountain Sickness; OR, odds ratio; weights are from random‐effects model.

**Figure S2:** Forest plot of associations between anemia and cognitive impairment: findings from a meta‐analysis. CIs, Confidence interval; OR, odds ratio; weights are from fixed‐effects model.

**Figure S3:** Forest plot of associations between OSA and cognitive impairment: findings from a meta‐analysis. CIs, Confidence interval; OR, odds ratio; OSA, Obstructive Sleep Apnoea; weights are from fixed‐effects model.

**Figure S4:** Forest plot of associations between COPD and cognitive impairment: findings from a meta‐analysis. CIs, Confidence interval; COPD, Chronic Obstructive Pulmonary Disease; OR, Odds ratio; weights are from fixed‐effects model.

**Figure S5:** Forest plot of associations between four disease: findings from a meta‐analysis. CIs, Confidence interval; OR, odds ratio; weights are from random‐effects model.

**Figure S6:** Funnel plot: association of four diseases with the prevalence of cognitive impairment (odds ratios, OR). Each point represents an individual study, plotted according to its effect size (OR) and corresponding standard error. The vertical line indicates the pooled effect estimate. Symmetry of the plot suggests a low risk of publication bias, whereas asymmetry may indicate potential bias. Egger's test was performed to statistically assess funnel plot asymmetry. OR, odds ratio.

**Figure S7:** Funnel plot: association of four diseases with cognitive function scores (standardized mean differences, SMD). Each point represents an individual study, plotted according to its effect size (SMD) and corresponding standard error. The vertical line indicates the pooled effect estimate. Symmetry of the plot suggests a low risk of publication bias, whereas asymmetry may indicate potential bias. Egger's test was performed to statistically assess funnel plot asymmetry. SMD, standardized mean difference.

**Table S1:** Search terms used in this systematic meta‐analysis.
**Table S2:** Cognitive domains, typical tests, and corresponding cognitive processes.
**Table S3:** Risk of bias evaluation based on the Newcastle‐Ottawa Scale (NOS).
**Table S4:** Meta‐analytic effects of CMS, anemia, OSA and COPD on cognitive impairment.
**Table S5:** Standardized mean differences (SMD) in cognitive performance by disease type (three‐level hierarchical model).
**Table S6:** Comparative **s**tandardized mean differences of cognitive domains by disease group.
**Table S7:** Standardized mean differences (SMD) in cognitive performance by disease type (two‐level hierarchical model).
**Table S8:** Standardized mean differences (SMD) in cognitive domains between experimental and control groups (two‐level hierarchical model).

## Data Availability

The data supporting this meta‐analysis are from previously published studies, which are all listed in the references. The synthesized dataset (extracted and compiled for analysis) is available from the corresponding author upon reasonable request.
