# Supplementary material for: Chronic Cerebral Hypoxia and Cognitive Impairment: A Systematic Review and Meta‐Analysis Based on Chronic Mountain Sickness, Anemia, Chronic Obstructive Pulmonary Disease, and Obstructive Sleep Apnea
Source: CNS Neurosci Ther. 2026 Apr 16;32(4):e70875. doi: 10.1002/cns.70875 (PMC13087434; doi:10.1002/cns.70875)
Supplement: Supplementary file 1 — Appendix S1: PRISMA 2020 main checklist. [file CNS-32-e70875-s007.docx]

# PRISMA 2020 Main Checklist

| **Topic** | **No.** | **Item** | **Location where item is reported** |
| --- | --- | --- | --- |
| **TITLE** |  |  |  |
| **Title** | 1 | Identify the report as a systematic review. | Title section on the first page of the document |
| **ABSTRACT** |  |  |  |
| **Abstract** | 2 | See the PRISMA 2020 for Abstracts checklist |  |
| **INTRODUCTION** |  |  |  |
| **Rationale** | 3 | Describe the rationale for the review in the context of existing knowledge. | Paragraphs 1-3 of the "Introduction" section |
| **Objectives** | 4 | Provide an explicit statement of the objective(s) or question(s) the review addresses. | Paragraph 4 of the "Introduction" section |
| **METHODS** |  |  |  |
| **Eligibility criteria** | 5 | Specify the inclusion and exclusion criteria for the review and how studies were grouped for the syntheses. | "Study selection" section (Inclusion Criteria 1-6, Exclusion Criteria 1-5) and "Operational definition" section (diagnostic criteria for the four diseases) in the "Methods" |
| **Information sources** | 6 | Specify all databases, registers, websites, organisations, reference lists and other sources searched or consulted to identify studies. Specify the date when each source was last searched or consulted. | "Literature search" section |
| **Search strategy** | 7 | Present the full search strategies for all databases, registers and websites, including any filters and limits used. | "Literature search" section |
| **Selection process** | 8 | Specify the methods used to decide whether a study met the inclusion criteria of the review, including how many reviewers screened each record and each report retrieved, whether they worked independently, and if applicable, details of automation tools used in the process. | "Literature search" section |
| **Data collection process** | 9 | Specify the methods used to collect data from reports, including how many reviewers collected data from each report, whether they worked independently, any processes for obtaining or confirming data from study investigators, and if applicable, details of automation tools used in the process. | "Data extraction" section |
| **Data items** | 10a | List and define all outcomes for which data were sought. Specify whether all results that were compatible with each outcome domain in each study were sought (e.g. for all measures, time points, analyses), and if not, the methods used to decide which results to collect. | Operational definition (exposure variables: CMS, Anemia, OSA, COPD; outcome variables: cognitive impairment, cognitive domains with standardized criteria) |
|  | 10b | List and define all other variables for which data were sought (e.g. participant and intervention characteristics, funding sources). Describe any assumptions made about any missing or unclear information. | Data extraction and quality assessment (extracted data: study origin, participant characteristics [age, sample size, sex distribution], diagnostic criteria, outcome assessment methods, adjusted covariates) |
| **Study risk of bias assessment** | 11 | Specify the methods used to assess risk of bias in the included studies, including details of the tool(s) used, how many reviewers assessed each study and whether they worked independently, and if applicable, details of automation tools used in the process. | Data extraction and quality assessment (Newcastle - Ottawa Scale (NOS) adapted; 2 reviewers rated independently, discrepancies resolved by a third) |
| **Effect measures** | 12 | Specify for each outcome the effect measure(s) (e.g. risk ratio, mean difference) used in the synthesis or presentation of results. | Statistical analyses (pooled results as odds ratios [ORs] and standardized mean differences [SMDs] with 95% CIs) |
| **Synthesis methods** | 13a | Describe the processes used to decide which studies were eligible for each synthesis (e.g. tabulating the study intervention characteristics and comparing against the planned groups for each synthesis (item 5)). | Statistical analyses (model selection based on heterogeneity; allowance for multiple independent effect sizes due to multi - tool assessments, multiple outcomes, time - point variations) |
|  | 13b | Describe any methods required to prepare the data for presentation or synthesis, such as handling of missing summary statistics, or data conversions. | "Statistical analyses" section |
|  | 13c | Describe any methods used to tabulate or visually display results of individual studies and syntheses. | "Statistical analyses" section |
|  | 13d | Describe any methods used to synthesize results and provide a rationale for the choice(s). If meta-analysis was performed, describe the model(s), method(s) to identify the presence and extent of statistical heterogeneity, and software package(s) used. | Statistical analyses (software: Stata 18.0, R) |
|  | 13e | Describe any methods used to explore possible causes of heterogeneity among study results (e.g. subgroup analysis, meta-regression). | Statistical analyses (subgroup analyses to identify heterogeneity sources) |
|  | 13f | Describe any sensitivity analyses conducted to assess robustness of the synthesized results. | Statistical analyses (leave - one - out sensitivity analysis to assess individual study impacts) |
| **Reporting bias assessment** | 14 | Describe any methods used to assess risk of bias due to missing results in a synthesis (arising from reporting biases). | Statistical analyses (Egger’s test with trim - and - fill adjustment if needed) |
| **Certainty assessment** | 15 | Describe any methods used to assess certainty (or confidence) in the body of evidence for an outcome. | "Statistical analyses" section |
| **RESULTS** |  |  |  |
| **Study selection** | 16a | Describe the results of the search and selection process, from the number of records identified in the search to the number of studies included in the review, ideally using a flow diagram. | Paragraphs 1 of the "Study selection and characteristics" section |
|  | 16b | Cite studies that might appear to meet the inclusion criteria, but which were excluded, and explain why they were excluded. | Paragraphs 1 of the "Study selection and characteristics" section |
| **Study characteristics** | 17 | Cite each included study and present its characteristics. | Paragraphs 1 of the "Study selection and characteristics" section |
| **Risk of bias in studies** | 18 | Present assessments of risk of bias for each included study. | Paragraphs 2 of the "Study selection and characteristics" section |
| **Results of individual studies** | 19 | For all outcomes, present, for each study: (a) summary statistics for each group (where appropriate) and (b) an effect estimate and its precision (e.g. confidence/credible interval), ideally using structured tables or plots. | "Associations between CMS, anemia, OSA, COPD and cognitive impairment" section and"Associations between CMS, anemia, OSA, COPD and cognitive function" section |
| **Results of syntheses** | 20a | For each synthesis, briefly summarise the characteristics and risk of bias among contributing studies. | "Associations between CMS, anemia, OSA, COPD and cognitive impairment" section and"Associations between CMS, anemia, OSA, COPD and cognitive function" section |
|  | 20b | Present results of all statistical syntheses conducted. If meta-analysis was done, present for each the summary estimate and its precision (e.g. confidence/credible interval) and measures of statistical heterogeneity. If comparing groups, describe the direction of the effect. | "Associations between CMS, anemia, OSA, COPD and cognitive impairment" section and"Associations between CMS, anemia, OSA, COPD and cognitive function" section |
|  | 20c | Present results of all investigations of possible causes of heterogeneity among study results. | Paragraphs 1-2 of the "Publication bias and sensitivity analysis" section |
|  | 20d | Present results of all sensitivity analyses conducted to assess the robustness of the synthesized results. | Paragraphs 3-4 of the "Publication bias and sensitivity analysis" section |
| **Reporting biases** | 21 | Present assessments of risk of bias due to missing results (arising from reporting biases) for each synthesis assessed. | Paragraphs 1-4 of the "Publication bias and sensitivity analysis" section |
| **Certainty of evidence** | 22 | Present assessments of certainty (or confidence) in the body of evidence for each outcome assessed. | Paragraphs 1-4 of the "Publication bias and sensitivity analysis" section |
| **DISCUSSION** |  |  |  |
| **Discussion** | 23a | Provide a general interpretation of the results in the context of other evidence. | "Mechanisms: Pathway Analysis from Hypoxia to Cognitive Impairment" section |
|  | 23b | Discuss any limitations of the evidence included in the review. | "Consensus, Controversies, and Significance of the Study" section |
|  | 23c | Discuss any limitations of the review processes used. | "Heterogeneity analysis: methodological limitations and biological interpretations" section |
|  | 23d | Discuss implications of the results for practice, policy, and future research. | "Clinical Implications: Translating Evidence into Practice" section and "Future Research Directions" section |
| **OTHER INFORMATION** |  |  |  |
| **Registration and protocol** | 24a | Provide registration information for the review, including register name and registration number, or state that the review was not registered. | "Protocol and registration" in the ‘’Methods"' |
|  | 24b | Indicate where the review protocol can be accessed, or state that a protocol was not prepared. | No review protocol was prepared |
|  | 24c | Describe and explain any amendments to information provided at registration or in the protocol. | There were no modifications to the registered or protocol information |
| **Support** | 25 | Describe sources of financial or non-financial support for the review, and the role of the funders or sponsors in the review. | "Funding" section |
| **Competing interests** | 26 | Declare any competing interests of review authors. | "Declaration of interest" section |
| **Availability of data, code and other materials** | 27 | Report which of the following are publicly available and where they can be found: template data collection forms; data extracted from included studies; data used for all analyses; analytic code; any other materials used in the review. | "Data Availability" section |

# PRIMSA Abstract Checklist

| **Topic** | **No.** | **Item** | **Reported?** |
| --- | --- | --- | --- |
| **TITLE** |  |  |  |
| **Title** | 1 | Identify the report as a systematic review. | Yes |
| **BACKGROUND** |  |  |  |
| **Objectives** | 2 | Provide an explicit statement of the main objective(s) or question(s) the review addresses. | Yes |
| **METHODS** |  |  |  |
| **Eligibility criteria** | 3 | Specify the inclusion and exclusion criteria for the review. | Yes |
| **Information sources** | 4 | Specify the information sources (e.g. databases, registers) used to identify studies and the date when each was last searched. | Yes |
| **Risk of bias** | 5 | Specify the methods used to assess risk of bias in the included studies. | Yes |
| **Synthesis of results** | 6 | Specify the methods used to present and synthesize results. | Yes |
| **RESULTS** |  |  |  |
| **Included studies** | 7 | Give the total number of included studies and participants and summarise relevant characteristics of studies. | Yes |
| **Synthesis of results** | 8 | Present results for main outcomes, preferably indicating the number of included studies and participants for each. If meta-analysis was done, report the summary estimate and confidence/credible interval. If comparing groups, indicate the direction of the effect (i.e. which group is favoured). | Yes |
| **DISCUSSION** |  |  |  |
| **Limitations of evidence** | 9 | Provide a brief summary of the limitations of the evidence included in the review (e.g. study risk of bias, inconsistency and imprecision). | Yes |
| **Interpretation** | 10 | Provide a general interpretation of the results and important implications. | Yes |
| **OTHER** |  |  |  |
| **Funding** | 11 | Specify the primary source of funding for the review. | Yes |
| **Registration** | 12 | Provide the register name and registration number. | Yes |

*From:* Page MJ, McKenzie JE, Bossuyt PM, Boutron I, Hoffmann TC, Mulrow CD, et al. The PRISMA 2020 statement: an updated guideline for reporting systematic reviews. MetaArXiv. 2020, September 14. DOI: 10.31222/osf.io/v7gm2. For more information, visit: <www.prisma-statement.org>
