# Supplementary figures and images for: Chronic Cerebral Hypoxia and Cognitive Impairment: A Systematic Review and Meta‐Analysis Based on Chronic Mountain Sickness, Anemia, Chronic Obstructive Pulmonary Disease, and Obstructive Sleep Apnea
Source: CNS Neurosci Ther. 2026 Apr 16;32(4):e70875. doi: 10.1002/cns.70875 (PMC13087434; doi:10.1002/cns.70875)

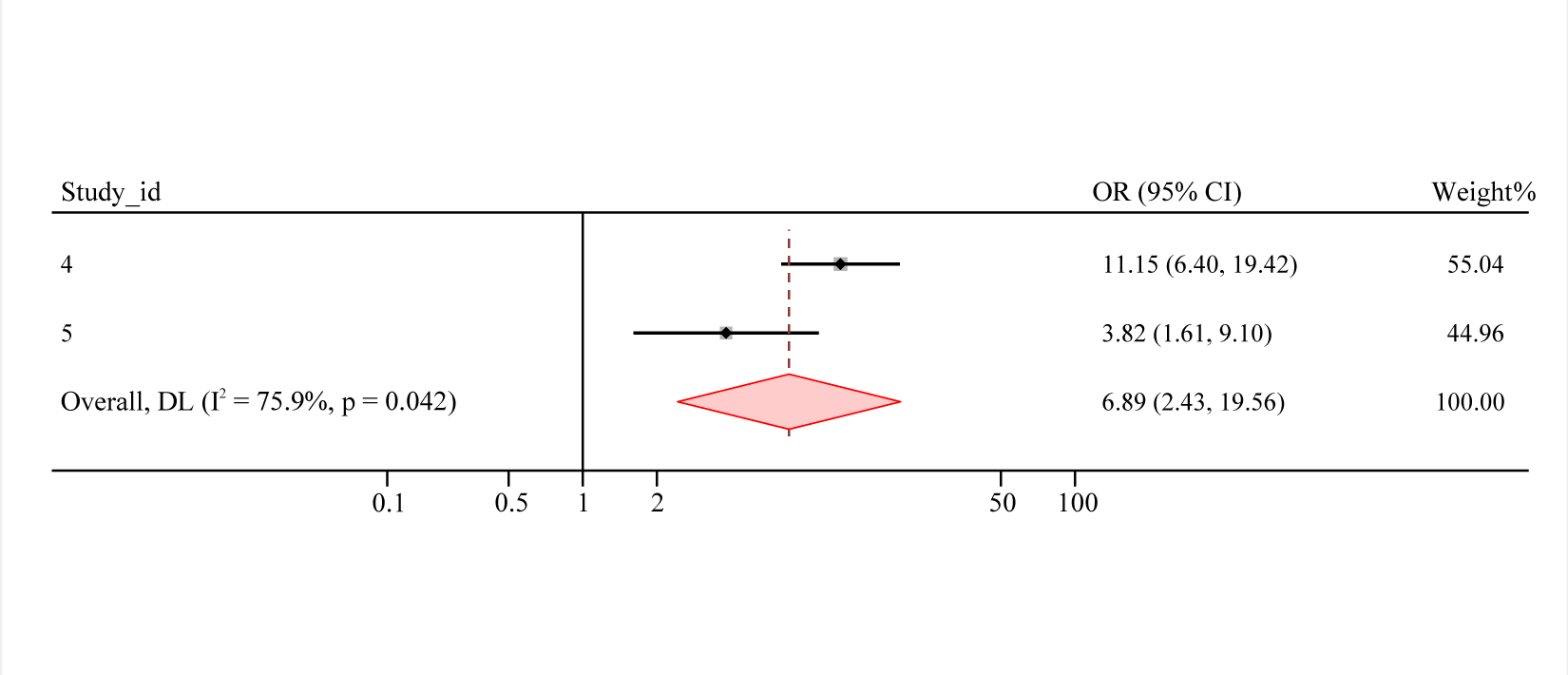

Supplement: Supplementary file 2 — Figure S1: Forest plot of associations between CMS and cognitive impairment: findings from a meta‐analysis. CIs, Confidence interval; CMS, Chronic Mountain Sickness; OR, odds ratio; weights are from random‐effects model. [file CNS-32-e70875-s003.tif]

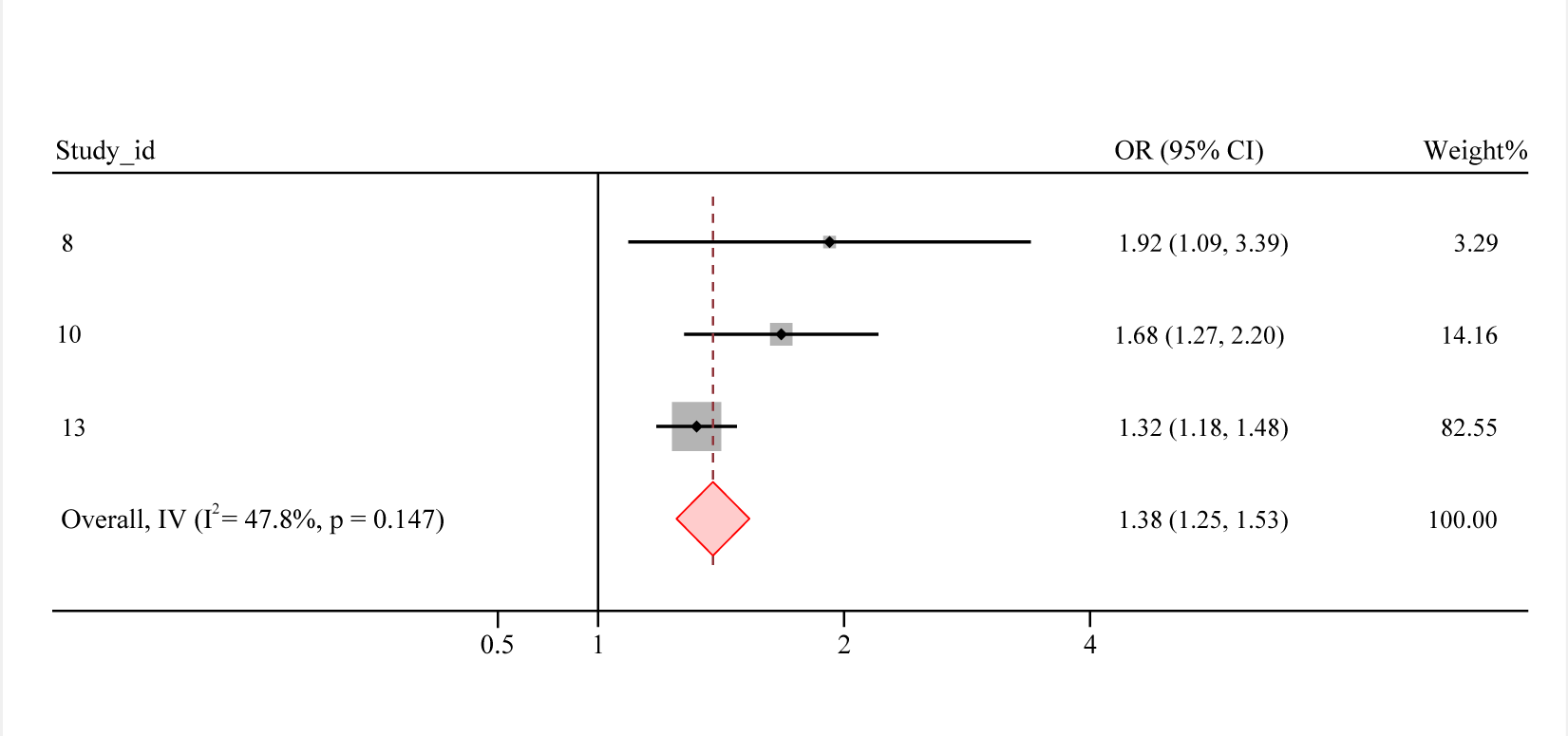

Supplement: Supplementary file 3 — Figure S2: Forest plot of associations between anemia and cognitive impairment: findings from a meta‐analysis. CIs, Confidence interval; OR, odds ratio; weights are from fixed‐effects model. [file CNS-32-e70875-s004.tif]

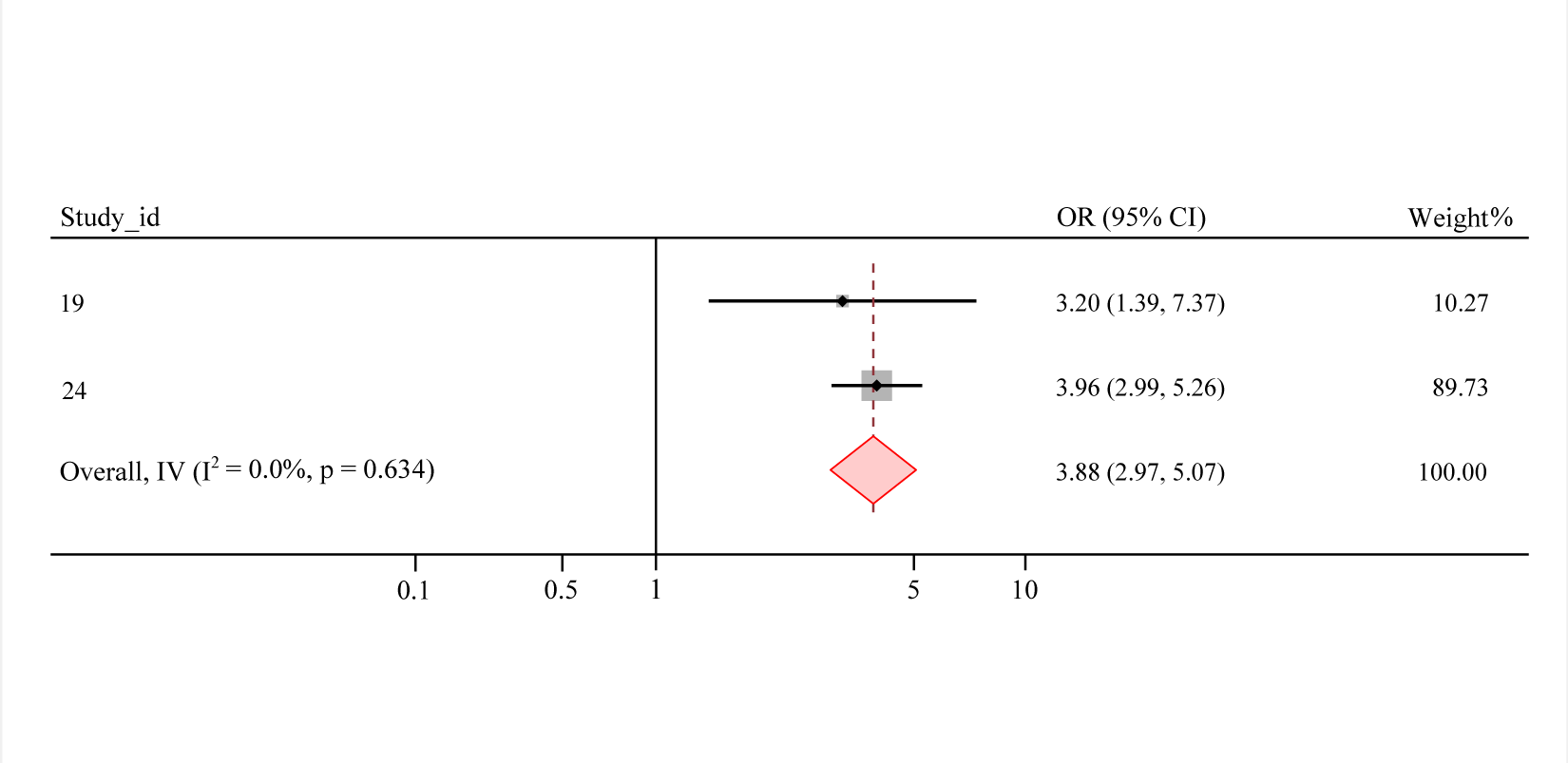

Supplement: Supplementary file 4 — Figure S3: Forest plot of associations between OSA and cognitive impairment: findings from a meta‐analysis. CIs, Confidence interval; OR, odds ratio; OSA, Obstructive Sleep Apnoea; weights are from fixed‐effects model. [file CNS-32-e70875-s002.tif]

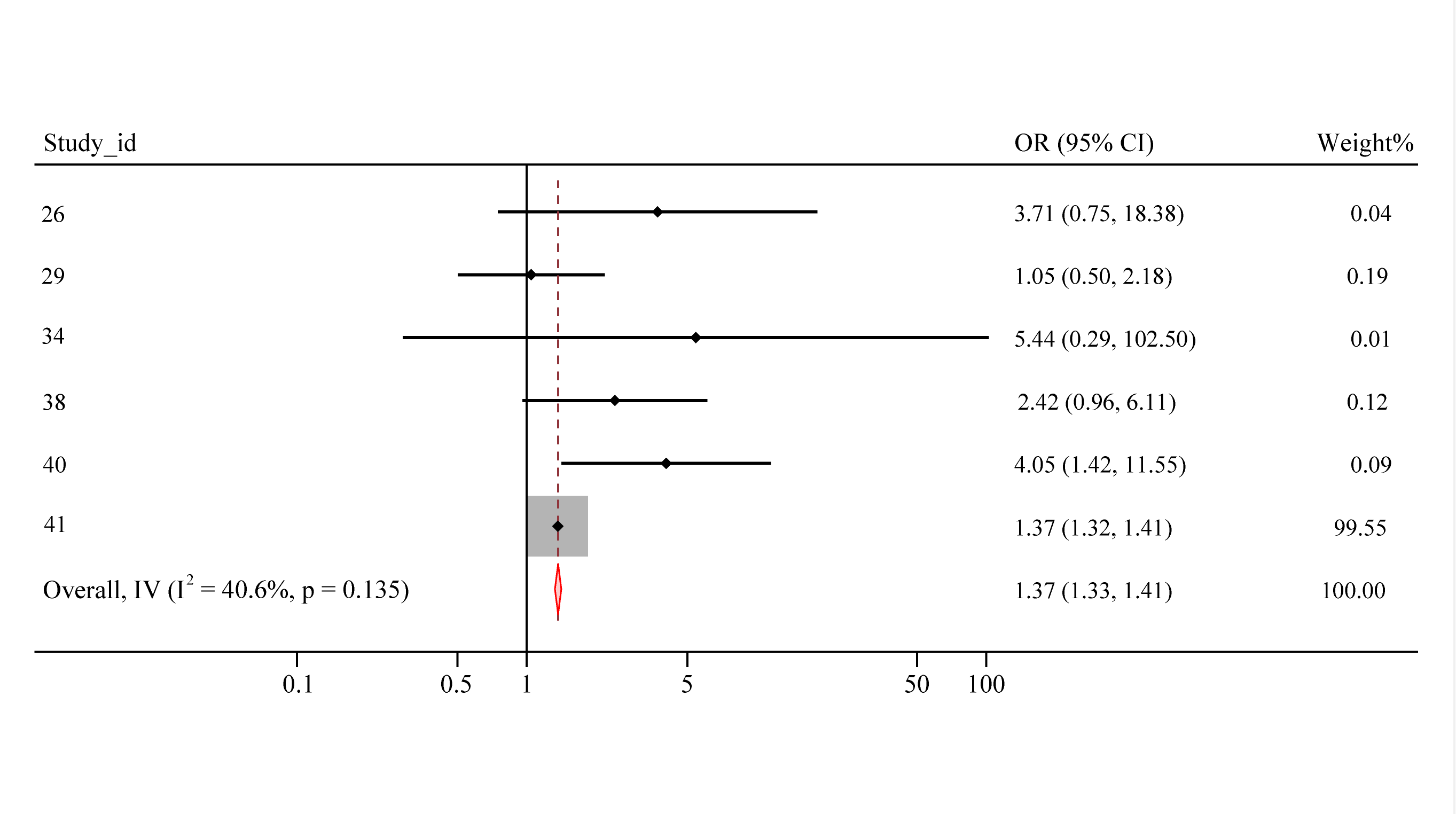

Supplement: Supplementary file 5 — Figure S4: Forest plot of associations between COPD and cognitive impairment: findings from a meta‐analysis. CIs, Confidence interval; COPD, Chronic Obstructive Pulmonary Disease; OR, Odds ratio; weights are from fixed‐effects model. [file CNS-32-e70875-s009.tif]

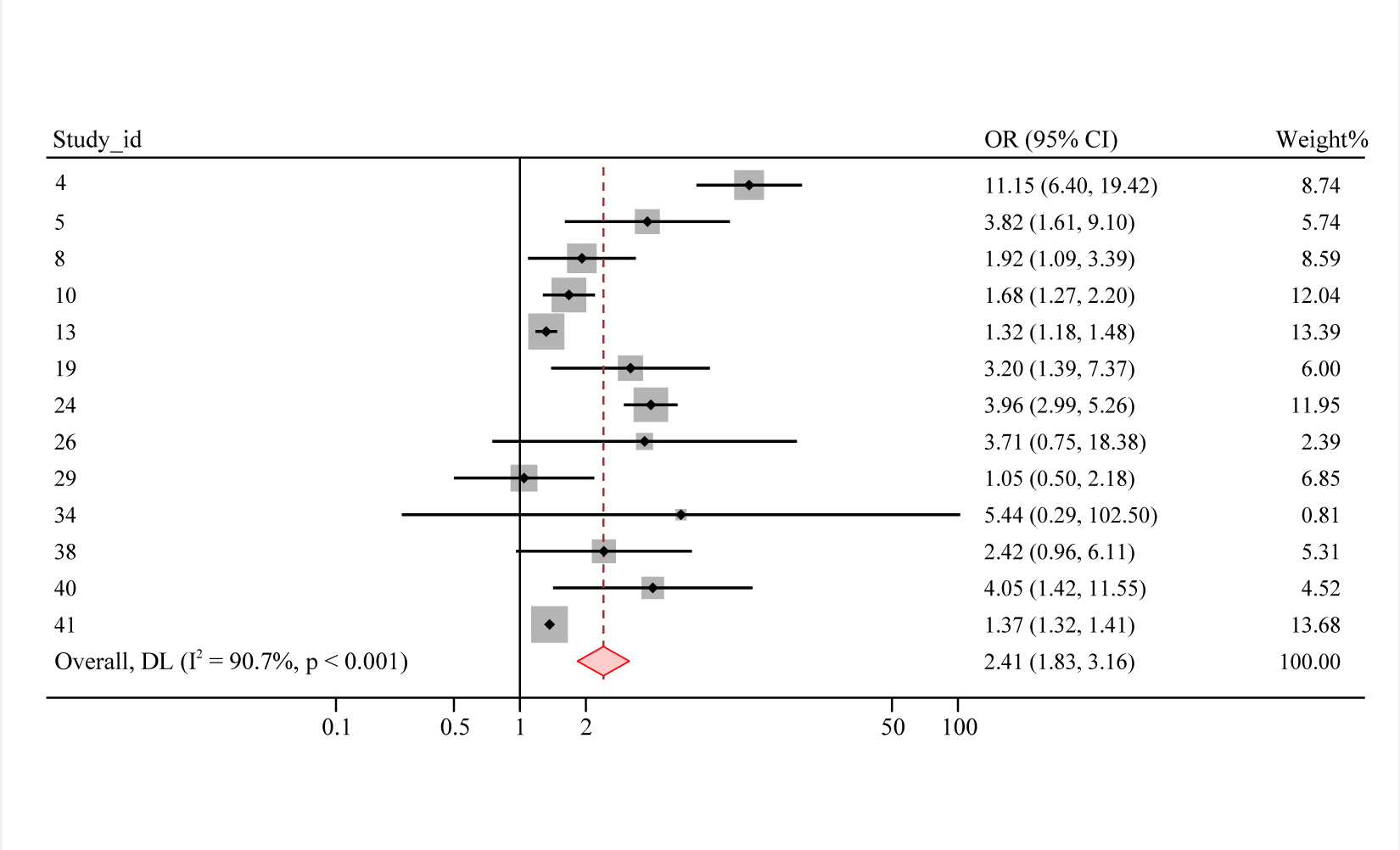

Supplement: Supplementary file 6 — Figure S5: Forest plot of associations between four disease: findings from a meta‐analysis. CIs, Confidence interval; OR, odds ratio; weights are from random‐effects model. [file CNS-32-e70875-s001.tif]

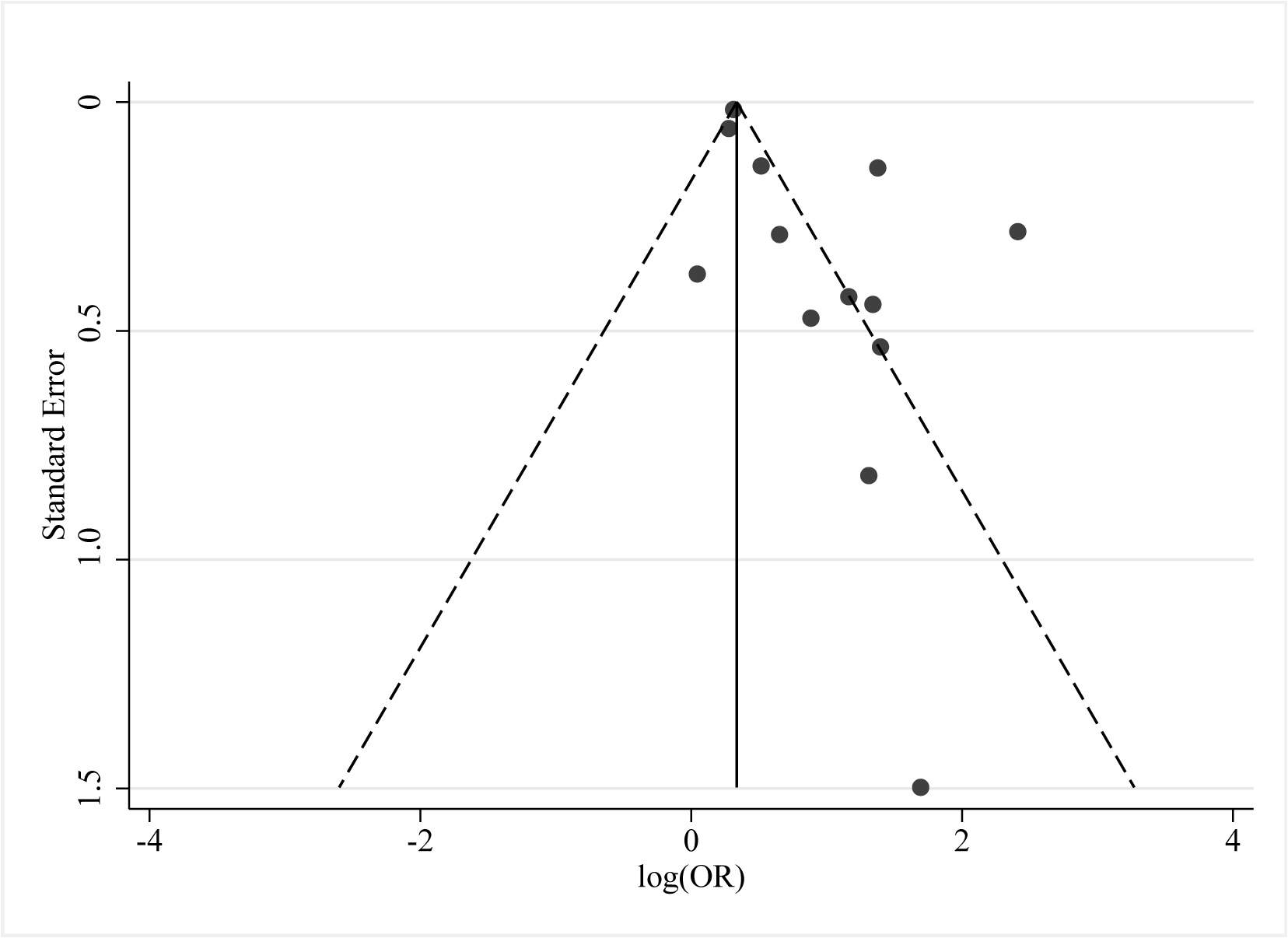

Supplement: Supplementary file 7 — Figure S6: Funnel plot: association of four diseases with the prevalence of cognitive impairment (odds ratios, OR). Each point represents an individual study, plotted according to its effect size (OR) and corresponding standard error. The vertical line indicates the pooled effect estimate. Symmetry of the plot suggests a low risk of publication bias, whereas asymmetry may indicate potential bias. Egger's test was performed to statistically assess funnel plot asymmetry. OR, odds ratio. [file CNS-32-e70875-s008.tif]

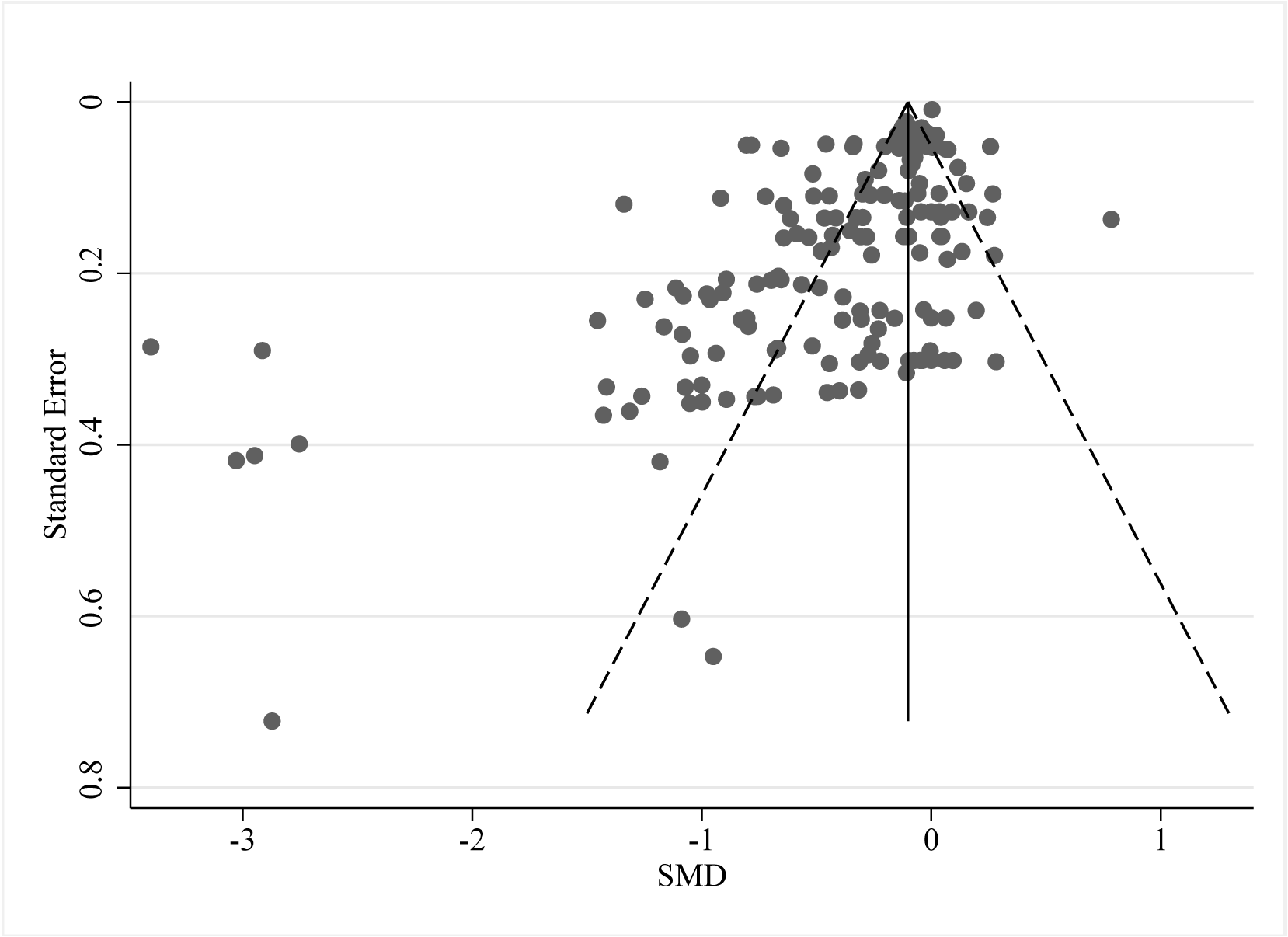

Supplement: Supplementary file 8 — Figure S7: Funnel plot: association of four diseases with cognitive function scores (standardized mean differences, SMD). Each point represents an individual study, plotted according to its effect size (SMD) and corresponding standard error. The vertical line indicates the pooled effect estimate. Symmetry of the plot suggests a low risk of publication bias, whereas asymmetry may indicate potential bias. Egger's test was performed to statistically assess funnel plot asymmetry. SMD, standardized mean difference. [file CNS-32-e70875-s006.tif]
