# Supplementary material for: Chronic Cerebral Hypoxia and Cognitive Impairment: A Systematic Review and Meta‐Analysis Based on Chronic Mountain Sickness, Anemia, Chronic Obstructive Pulmonary Disease, and Obstructive Sleep Apnea
Source: CNS Neurosci Ther. 2026 Apr 16;32(4):e70875. doi: 10.1002/cns.70875 (PMC13087434; doi:10.1002/cns.70875)
Supplement: Supplementary file 9 — Table S1: Search terms used in this systematic meta‐analysis. Table S2: Cognitive domains, typical tests, and corresponding cognitive processes. Table S3: Risk of bias evaluation based on the Newcastle‐Ottawa Scale (NOS). Table S4: Meta‐analytic effects of CMS, anemia, OSA and COPD on cognitive impairment. Table S5: Standardized mean differences (SMD) in cognitive performance by disease type (three‐level hierarchical model). Table S6: Comparative standardized mean differences of cognitive domains by disease group. Table S7: Standardized mean differences (SMD) in cognitive performance by disease type (two‐level hierarchical model). Table S8: Standardized mean differences (SMD) in cognitive domains between experimental and control groups (two‐level hierarchical model). [file CNS-32-e70875-s005.docx]

Supplementary Table 1. Search terms used in this systematic meta-analysis.

| **Keywords** | **Search terms** |
| --- | --- |
| Cognitive Impairment | Dysfunction, Cognitive OR Dysfunctions, Cognitive OR Cognitive Disorder OR Cognitive Disorders OR Disorder, Cognitive OR Disorders, Cognitive OR Cognitive Impairments OR Cognitive Dysfunction OR Impairment, Cognitive OR Impairments, Cognitive OR Mild Cognitive Impairment OR Cognitive Impairment, Mild OR Cognitive Impairments, Mild OR Impairment, Mild Cognitive OR Impairments, Mild Cognitive OR Mild Cognitive Impairments OR Cognitive Decline OR Cognitive Declines OR Decline, Cognitive OR Declines, Cognitive OR Mental Deterioration OR Deterioration, Mental OR Deteriorations, Mental OR Mental Deteriorations |
| Altitude | Altitudes |
| Anemia | Anemias |
| Sleep Apnea, Obstructive | Apneas, Obstructive Sleep OR Obstructive Sleep Apneas OR Sleep Apneas, Obstructive OR Apnea, Obstructive Sleep OR Sleep Apnea Hypopnea Syndrome OR Obstructive Sleep Apnea Syndrome OR Obstructive Sleep Apnea OR Syndrome, Obstructive Sleep Apnea OR Syndrome, Sleep Apnea, Obstructive OR Sleep Apnea Syndrome, Obstructive OR OSAHS OR Upper Airway Resistance Sleep Apnea Syndrome OR Syndrome, Upper Airway Resistance, Sleep Apnea |
| Pulmonary Disease,  Chronic Obstructive | Chronic Obstructive Pulmonary Diseases OR COPD OR Chronic Obstructive Lung Disease OR Chronic Obstructive Pulmonary Disease OR COAD OR Chronic Obstructive Airway Disease OR Airflow Obstruction, Chronic OR Airflow Obstructions, Chronic OR Chronic Airflow Obstructions OR Chronic Airflow Obstruction Pulmonary Disease, Chronic Obstructive OR Chronic Obstructive Pulmonary Diseases OR COPD OR Chronic Obstructive Lung Disease OR Chronic Obstructive Pulmonary Disease OR COAD OR Chronic Obstructive Airway Disease OR Airflow Obstruction, Chronic OR Airflow Obstructions, Chronic OR Chronic Airflow Obstructions OR Chronic Airflow Obstruction |

Supplementary Table 2. Cognitive domains, Typical tests, and Corresponding cognitive processes.

| **Cognitive domain** | **Example tasks** | **Key cognitive processes** |
| --- | --- | --- |
| Attention working memory | Digit Span; Continuous Performance Test (CPT); Paced Auditory Serial Addition Test (PASAT); N-back Task; Attention Network Test(ANT); D2 Test; Conners Continuous; Performance Test; Corsi Blocks Forward; Counting Span | sustained attention, selective attention, holding & manipulating info |
| Executive functions | Trail Making Test Part B (TMT-B); Stroop Test; Wisconsin Card Sorting Test (WCST); Auditory Recognition Reaction Time(ACRT); Plus Minus; Behavioural Assessment of the Dysexecutive Syndrome (BADS) Zoo Map, Key Search; Dual Task; Abstraction(Montreal Cognitive Assessment, MoCA); Groton Maze Learning; Stroop Color and Word Test (SCWT) Card III; Category Switching Test Part C (CST-C); Abstract Verbal Reasoning | cognitive flexibility/shifting, inhibitory control, planning & problem-solving |
| Memory | Rey Auditory Verbal Learning Test (RAVLT); Rey Complex Figure Test Recall; Hopkins Verbal Learning Test (HVLT); Wechsler Memory Scale (WMS); CERAD Word Memory Test; 8 - Word List from The Nuremberg Geriatric Inventory; Orientation,Procedural Memory,Delayed Recall,Memory Registration & Immediate Recall(Multi Domain Cognitive Screening Test,MDCST); Delayed Verbal Memory(DVBM); Immediate Verbal Memory(IVBM); Benton Visual Retention Test; Consortium to Establish a Registry for Alzheimer’s Disease(CERAD); word memory test; Letter Memory Task; Visual Verbal Learning Test(VVLT) Trial 1; Interference Memory 10”/30”; Logical Memory Delayed Recall; Rey Osterreith Complex Figure Test(RCFT); Autobiographical Memory Test; Digit-Symbol Pairs; Oktem Verbal Memory Processes Test(OVMPT); Visual Verbal Learning Test; Orientation,Registration,Recall(mini-mental state examination,MMSE) | immediate & delayed recall, recognition, episodic memory encoding and retrieval |
| Language | Boston Naming Test (BNT); Verbal Fluency Test (FAS / Category); Token Test; Language(MDCST); Animal Semantic Fluency Test; Naming,Language(MoCA); Language(MMSE); Phonetic Fluency; Letter Fluency; Category Fluency; Word Fluency; Animal Naming; Word Fluency Test(WFT) | word retrieval, expressive & receptive language, semantic memory |
| Visuospatial ability | Rey-Osterrieth Complex Figure; Benton Judgment of Line Orientation; Clock Drawing Test; Pursuit Aiming Test; Progressive Raven Matrices; Corsi Cubes; Clock Time Perception; Standard Progressive Matrices(SPM); Overlapping Pictures; Spontaneous/Copy Drawing; Ideative/Ideomotor Praxis; Purdue Pegboard(PPB) Test | spatial perception, constructional ability, mental rotation |

(continued on next page)

Supplementary Table 2.(continued)

| **Cognitive domain** | **Example tasks** | **Key cognitive processes** |
| --- | --- | --- |
| Processing speed | Trail Making Test Part A (TMT-A); Symbol Digit Modalities Test (SDMT); Digit Symbol Substitution; Auditory Simple Reaction Time(ASRT); Letter-Digit Substitution Test (LDST); Stroop Color and Word Test Card I; Digit Symbol Coding Category Switching Test Part A (CST-A); Detection Speed; Identification Speed; Digital Symbol Test; Visual Reaction Time; Audible Reaction Time | speed of information processing, psychomotor speed |
| Global cognition | MMSE、MoCA、MDCST、BKSCA、RSPM、Episodic Memory and TICS score | integration of multiple cognitive domains to assess overall cognitive function. |

Supplementary Table 3. Risk of Bias Evaluation Based on the Newcastle-Ottawa Scale (NOS).

| **Study** | **Selection** | **Comparability** | **Outcome** | **Total score** |
| --- | --- | --- | --- | --- |
| Chen,2019 | 3 | 2 | 3 | 8 |
| Das, 2018 | 3 | 1 | 3 | 6 |
| Gao, 2015 | 3 | 1 | 3 | 7 |
| Hota, 2012 | 3 | 2 | 2 | 7 |
| Zhang, 2022 | 3 | 1 | 2 | 6 |
| Beydoun, 2020 | 3 | 2 | 3 | 8 |
| Deal, 2009 | 3 | 2 | 3 | 8 |
| Dlugaj, 2016 | 3 | 2 | 3 | 8 |
| Karismaz,2024 | 2 | 1 | 2 | 5 |
| Marzban, 2021 | 3 | 2 | 2 | 7 |
| Qin, 2019 | 3 | 2 | 2 | 7 |
| Valladão Júnior, 2020 | 3 | 2 | 2 | 7 |
| Zamboni,2006 | 2 | 2 | 2 | 6 |
| Badr,2023 | 3 | 1 | 2 | 6 |
| Borges,2013 | 3 | 2 | 2 | 7 |
| Buratti,2017 | 3 | 2 | 2 | 7 |
| Cavuoto,2023 | 3 | 2 | 2 | 7 |
| Chen,2011 | 3 | 1 | 2 | 6 |
| Kim,2017 | 3 | 2 | 2 | 7 |
| Kong,2021 | 3 | 2 | 2 | 7 |
| Lutsey, 2016 | 3 | 2 | 3 | 8 |
| Macchitella, 2024 | 3 | 2 | 2 | 7 |
| Mekky, 2022 | 3 | 2 | 2 | 7 |
| Yerlikaya, 2018 | 3 | 1 | 2 | 6 |
| Bratek, 2015 | 3 | 1 | 2 | 6 |
| Cleutjens, 2017 | 3 | 2 | 3 | 8 |
| Crisan, 2014 | 3 | 1 | 2 | 6 |
| Dodd, 2013 | 3 | 2 | 2 | 7 |
| Isoaho, 1996 | 3 | 1 | 2 | 6 |
| Klein, 2010 | 3 | 2 | 2 | 7 |
| Kozora, 1999 | 3 | 2 | 2 | 7 |
| Krishnamurthy, 2019 | 3 | 2 | 2 | 7 |
| Li, 2013 | 3 | 2 | 2 | 7 |
| Özge, 2006 | 3 | 1 | 2 | 6 |
| Pierobon, 2018 | 3 | 2 | 2 | 7 |

(continued on next page)

Supplementary Table 3. (continued)

| **Study** | **Selection** | **Comparability** | **Outcome** | **Total score** |
| --- | --- | --- | --- | --- |
| Salık, 2007 | 3 | 2 | 2 | 7 |
| Fekri, 2017 | 3 | 2 | 2 | 7 |
| Thakur, 2010 | 3 | 2 | 2 | 7 |
| Xiao, 2022 | 3 | 2 | 3 | 8 |
| Villeneuve, 2012 | 3 | 2 | 2 | 7 |
| Siraj, 2021 | 3 | 2 | 3 | 8 |

Supplementary Table 4. Meta-analytic Effects of CMS,Anemia,OSA and COPD on Cognitive Impairment.

| **Disease** | **k** | **Effects model** | **Sample** | **Pooled results OR (95 % CIs)** | **Heterogeneity** | |
| --- | --- | --- | --- | --- | --- | --- |
|  |  |  |  |  | **I²** | ***P*-value** |
| CMS | 2 | random | 1,797 | 6.892(2.429,19.559)*** | 75.9% | 0.042 |
| Anemia | 3 | fixed | 18,318 | 1.382(1.247,1.532)*** | 47.8% | 0.147 |
| OSA | 2 | fixed | 1,839 | 3.879(2.969,5.066)*** | 0.00% | 0.634 |
| COPD | 6 | fixed | 310,000 | 1.370(1.327,1.414)*** | 40.6% | 0.135 |
| Total disease | 13 | random | 332,598 | 2.407(1.833,3.161)*** | 90.7% | <0.001 |

Abbreviations: CMS, Chronic Mountain Sickness; COPD, Chronic Obstructive Pulmonary Disease; OSA, Obstructive Sleep Apnoea; k, number of studies; OR, odds ratio; CIs, Confidence interval; Significance levels: **p*<0.05; ***p*<0.01; ****p*<0.001.

Supplementary Table 5. Standardized Mean Differences (SMD) in Cognitive Performance by Disease Type (Three-Level Hierarchical Model).

| **Disease** | **N** | **SMD** | **95%CI** | | **z** | ***p*** |
| --- | --- | --- | --- | --- | --- | --- |
|  |  |  | **low** | **up** |  |  |
| CMS | 27 | -0.5577 | -1.0037 | -0.1116 | -2.4505 | **0.0143** |
| Anemia | 14 | -0.1504 | -0.5734 | 0.2727 | -0.6967 | 0.4860 |
| OSA | 72 | -0.4796 | -0.7944 | -0.1648 | -2.9858 | **0.0028** |
| COPD | 59 | -0.5749 | -0.8563 | -0.2935 | -4.0039 | **< 0.0001** |

Abbreviations:CMS, Chronic Mountain Sickness; COPD, Chronic Obstructive Pulmonary Disease; OSA, Obstructive Sleep Apnoea; N, number of effect sizes; SMD, standardized mean differences; CIs, Confidence interval; Bold *p*-values are statistically significant (*p* < 0.05); Analyses were conducted using a random-effects model.

Supplementary Table 6.Comparative **S**tandardized Mean Differences of Cognitive Domains by Disease Group.

|  | **N** | **Total diseases**  **SMD(95%CIs)ᵃ** | **CMS**  **SMD(95%CIs)ᵃ** | **Anemia**  **SMD(95%CIs)ᵃ** | **OSA**  **SMD(95%CIs)ᵃ** | **COPD**  **SMD(95%CIs)ᵃ** |
| --- | --- | --- | --- | --- | --- | --- |
|  |  |  |  |  |  |  |
| **Executive functions** | 25 | -0.5154  (-0.7885,-0.2422)*** | -0.6936  (-1.7770,0.3899) | -0.3260  (-1.0627,0.4107) | -0.3623  (-0.8065,0.0818) | -0.7418  (-1.1942,-0.2894)** |
| **Memory** | 45 | -0.4231  (-0.6536,-0.1926)*** | -0.7070  (-1.2536,-0.1604)* | -0.0624  (-0.6137,0.4890) | -0.4520  (-0.8699,-0.0341)* | -0.4004  (-0.8085,0.0078) |
| **Processing speed** | 19 | -0.5120  (-0.8162,-0.2078)*** | -1.2046 (-1.9588,-0.4505)** | -0.2536  (-1.3065,0.7993) | -0.5257  (-1.0802,0.0287) | -0.3558  (-0.8398,0.1282) |
| **Language** | 24 | -0.3872  (-0.6541,-0.1202)** | -0.1471  (-0.8811,0.5869) | -0.2010  (-0.9287,0.5266) | -0.4698  (-0.9109,-0.0287)* | -0.3897  (-0.8654,0.0860) |
| **Attention working memory** | 15 | -0.2000  (-0.5095,0.1096) | -0.0319  (-1.0097,0.9458) | NA | -0.2097  (-0.6862,0.2668) | -0.3121  (-0.8067,0.1825) |
| **Visuospatial ability** | 12 | -0.6352  (-1.0040,-0.2665)*** | -0.5319  (-1.5961,0.5322) | NA | -0.0658  (-0.7482,0.6166) | -1.0359  (-1.5607,-0.5111)*** |
| **Global cognition** | 31 | -0.5465  (-0.7732,-0.3197)*** | -0.4194  (-0.9729,0.1341) | -0.1314  (-0.6774,0.4146) | -0.7131  (-1.1266,-0.2995)*** | -0.6662  (-1.0596,-0.2727)*** |

Abbreviations: CMS, Chronic Mountain Sickness; COPD, Chronic Obstructive Pulmonary Disease; OSA, Obstructive Sleep Apnoea; N,

number of effect sizes; SMD, standardized mean differences; CIs, Confidence interval; NA, not applicable.ᵃ the random-effects model

wasutilized; Significance levels: **p*<0.05;***p*<0.01; ****p*<0.001.

Supplementary Table 7. Standardized Mean Differences (SMD) in Cognitive Performance by Disease Type (Two-Level Hierarchical Model).

| **Disease** | **SMD** | **95%CI** | | **z** | ***p*** |
| --- | --- | --- | --- | --- | --- |
|  |  | **low** | **up** |  |  |
| CMS | -0.5611 | -1.0035 | -0.1188 | -2.4861 | **0.0129** |
| Anemia | -0.1522 | -0.5694 | 0.2650 | -0.7149 | 0.4747 |
| OSA | -0.4701 | -0.7818 | -0.1583 | -2.9555 | **0.0031** |
| COPD | -0.5715 | -0.8495 | -0.2936 | -4.0301 | **< 0.0001** |

Abbreviations: CMS, Chronic Mountain Sickness; COPD, Chronic Obstructive Pulmonary Disease; OSA, Obstructive Sleep Apnoea; SMD, standardized mean differences; CI, confidence interval; Bold *p*-values are statistically significant (*p* < 0.05); Analyses were conducted using a random-effects.

Supplementary Table 8. Standardized Mean Differences (SMD) in Cognitive Domains Between Experimental and Control Groups (Two-Level Hierarchical Model).

| **Cognitive domain** | **SMD** | **95%CI** | | **z** | ***p*** |
| --- | --- | --- | --- | --- | --- |
|  |  | **low** | **up** |  |  |
| Executive Functions | -0.5272 | -0.7685 | -0.2859 | -4.2822 | **< 0.0001** |
| Memory | -0.4629 | -0.6681 | -0.2576 | -4.4193 | **< 0.0001** |
| Processing Speed | -0.5152 | -0.7695 | -0.2609 | -3.9714 | **< 0.0001** |
| Language | -0.4267 | -0.6611 | -0.1922 | -3.5672 | **0.0004** |
| Attention Working Memory | -0.2075 | -0.4843 | 0.0693 | -1.4694 | 0.1417 |
| Visuospatial Ability | -0.6063 | -0.9029 | -0.3096 | -3.9714 | **< 0.0001** |
| Global Cognition | -0.5198 | -0.7315 | -0.3082 | -4.8144 | **< 0.0001** |

Abbreviations: SMD, standardized mean differences; CI, confidence interval; Bold p-values are statistically significant (*p* < 0.05); Analyses were conducted using a random-effects.
